# The Versatility of Pomegranate: From Phytochemical Diversity to Antimicrobial and Translational Applications

**DOI:** 10.3390/microorganisms14091948

**Published:** 2026-09-03

**Authors:** Daniela Sateriale, Giuseppina Forgione, Paola Salvatore, Caterina Pagliarulo

**Affiliations:** 1 Department of Science and Technology, University of Sannio, 82100 Benevento, Italy; sateriale@unisannio.it (D.S.); gforgione@unisannio.it (G.F.); 2 Department of Molecular Medicine and Medical Biotechnologies, University of Naples Federico II, 80100 Naples, Italy; psalvato@unina.it; 3 CEINGE-Biotecnologie Avanzate Franco Salvatore s.c.ar.l., 80100 Naples, Italy

**Keywords:** *Punica granatum*, pomegranate by-products, phytochemical diversity, green extraction, antimicrobial activity, multidrug resistance, biofilm, antimicrobial synergy, microbiota modulation, circular bioeconomy

## Abstract

Pomegranate is increasingly being recognized as a versatile source of bioactive compounds with antimicrobial, antioxidant, anti-inflammatory, and microbiota-modulating potential. Beyond the edible arils and juice, peel, seeds, leaves, flowers, and other agro-industrial by-products are rich in ellagitannins, particularly punicalagins, as well as ellagic acid, gallic acid, flavonoids, anthocyanins, fatty acids, and related phytochemicals. Their recovery depends strongly on plant fraction, cultivar, solvent, and extraction technology, including conventional hydroalcoholic extraction, ultrasound- and microwave-assisted processes, high-pressure treatments, and enzyme-assisted methods. Pomegranate-derived preparations exhibit activity against Gram-positive and Gram-negative bacteria, fungi, multidrug-resistant pathogens, and microbial biofilms, while selected compounds may enhance the efficacy of conventional antimicrobials. Emerging evidence also indicates bidirectional interactions with microbial communities, including microbial biotransformation of ellagitannins into urolithins and modulation of beneficial taxa and microbial metabolites. This narrative review integrates agronomic and phytochemical diversity, extraction and standardization strategies, mechanisms of antimicrobial action, synergistic interactions, microbiota-related effects, and applications in food preservation, biomedicine, animal nutrition, agriculture, and environmental sustainability. Key translational limitations include compositional variability, methodological heterogeneity, insufficient standardization, limited in vivo validation, and scarce evidence from realistic application models and clinical studies. Addressing these gaps is essential for developing safe, reproducible, and scalable pomegranate-derived preparations.

## 1. Introduction

**Figure 1:**
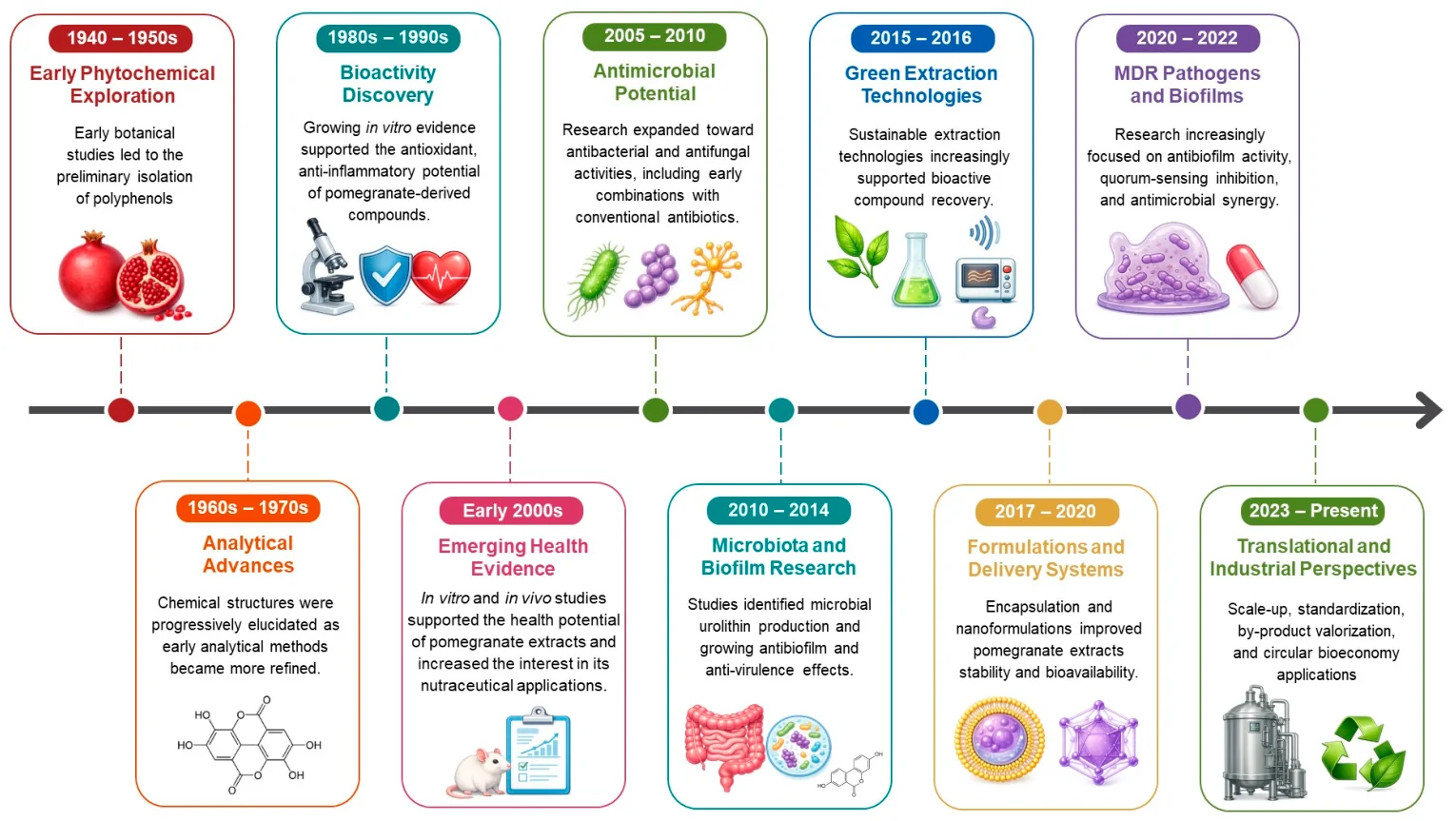
Evolution of pomegranate research and applications: timeline of major milestones and scientific evidence.

For centuries, pomegranate (*Punica granatum* L.) has held a prominent place in the diet, traditional medicine, and cultural practices of societies across Western and Central Asia, the Middle East, and the Mediterranean basin [1]. Although arils and juice are its best-known products, peel, seeds, leaves, flowers, bark, and roots have also been used for dietary and medicinal purposes. Modern pomegranate research has progressively moved from botanical, compositional, and phytochemical characterization toward the investigation of its biological and microbiological potential (Figure 1). Early analytical studies identified hydrolysable tannins, anthocyanins, phenolic acids, flavonoids, fatty acids, and sterols, providing the chemical basis for subsequent studies on antimicrobial and other biological activities [2,3,4]. Particular attention has increasingly been devoted to antibacterial, antifungal, antibiofilm, and resistance-modifying effects, including activity against oral pathogens, foodborne microorganisms, ESKAPE bacteria, and multidrug-resistant isolates [5,6,7,8,9]. In parallel, the ability of intestinal microorganisms to transform ellagitannins and ellagic acid into urolithins has highlighted the bidirectional relationship between pomegranate-derived compounds and microbial communities [10,11]. More recently, green extraction technologies, microbial bioprocessing, extract standardization, and by-product valorization have been developed to improve the recovery and reproducibility of bioactive fractions [12,13,14]. Importantly, the composition and functionality of pomegranate-derived preparations are strongly influenced by cultivar, geographical origin, agronomic conditions, plant fraction, processing, and extraction method [15,16]. These sources of variability are particularly relevant from a microbiological perspective because they can substantially affect antimicrobial potency, microbial selectivity, antibiofilm activity, interactions with antimicrobial-resistant strains, and the overall reproducibility of biological responses. Current research therefore increasingly aims to translate this phytochemical diversity into standardized, safe, selective, and scalable preparations for microbiologically relevant applications in food, biomedical, veterinary, agricultural, and environmental contexts.

Despite the extensive literature on *Punica granatum* and its derived products, current knowledge remains fragmented across agronomy, phytochemistry, microbiology, food science, nutrition, pharmacology, and process engineering. Previous reviews have provided valuable syntheses of the general health-promoting properties of pomegranate [4], its applications in food products and human nutrition [17], the influence of cultivar and plant fraction on bioactivity [15], the recovery and revalorization of processing by-products through green technologies [13], or the phytochemistry, extraction, and applications of pomegranate peel [18]. However, these complementary perspectives have rarely been integrated into a microbiology-oriented framework connecting plant source, extraction and chemical composition with microbial target, resistance phenotype, antimicrobial susceptibility, biofilm behaviour, microbial biotransformation, and microbiota modulation. In particular, comparatively limited attention has been devoted to multidrug-resistant pathogens, mature biofilms, synergistic interactions with conventional antimicrobials, microbial transformation of pomegranate phytochemicals, and selective effects on complex microbial communities. These gaps are particularly relevant from a microbiological perspective, since variability in plant source, extraction conditions, chemical composition, microbial strain, and experimental methodology can markedly affect the reproducibility and interpretation of antimicrobial outcomes.

The novelty of the present review therefore lies in integrating the previously fragmented aspects of pomegranate research within a single microbiology-oriented framework that links source and processing variables to antimicrobial activity, resistance-related responses, biofilm modulation, microbial biotransformation, and microbiota effects. Accordingly, the specific aim of this review is to provide an integrated source-to-application analysis of *P. granatum* that places its interactions with microorganisms at the centre of the discussion, linking botanical and agronomic diversity with biomass composition, conventional and green extraction technologies, microbial bioprocessing, phytochemical characterization, and extract standardization. It critically examines antimicrobial and antibiofilm activities, including effects against multidrug-resistant microorganisms and interactions with conventional antimicrobials, together with microbial biotransformation and microbiota-modulating effects. Antioxidant and anti-inflammatory activities are considered where they contribute to the interpretation of these microbiologically relevant applications. Particular emphasis is placed on the relationships among plant source, processing conditions, chemical composition, microbial target, and biological response. The review further provides an updated evaluation of applications in food preservation, biomedicine, animal nutrition, agriculture, environmental protection, and advanced delivery systems, while considering safety, regulatory requirements, industrial scale-up, and market translation. A distinctive feature of this approach is that microorganisms are considered not only as targets of pomegranate-derived antimicrobials, but also as mediators of phytochemical biotransformation and as components of complex host-associated and environmental microbial communities. By integrating microbial inhibition, microbial transformation, microbiota modulation, and circular biomass valorization, this review moves beyond a descriptive catalogue of biological activities and identifies the conditions required to develop standardized, selective, sustainable, and technologically viable pomegranate-derived preparations.

## 2. Methodology

This manuscript was designed as a structured narrative review aimed at critically synthesizing current knowledge on *Punica granatum* L. as a source of bioactive compounds, with particular emphasis on phytochemical diversity, extraction and bioprocessing strategies, antimicrobial and antibiofilm activities, interactions with conventional antimicrobials, microbiota modulation, and emerging applications in food, biomedical, veterinary, agricultural, and environmental fields. As a narrative review, this work did not follow a formal systematic-review protocol, and no quantitative meta-analysis was performed. Accordingly, a PRISMA-based selection process and formal risk-of-bias scoring were not prospectively applied, and a systematic numerical flow of records retrieved, screened, excluded, and included was not recorded. Nevertheless, a structured and criteria-based selection strategy was used to improve transparency and minimize the risk of arbitrary study selection.

A comprehensive literature search was conducted using PubMed, Scopus, Web of Science, and Google Scholar. The final search in all databases was performed on 31 July 2026. The search strategy combined terms related to the plant species and its different matrices with keywords referring to extraction technologies, chemical composition, biological activities, microbial interactions, and translational applications. The main search terms included the following: “*Punica granatum*”, “pomegranate”, “pomegranate peel”, “pomegranate seed”, “pomegranate leaf”, “pomegranate flower”, “pomegranate by-products”, “agro-industrial waste”, “phytochemicals”, “polyphenols”, “ellagitannins”, “punicalagin”, “ellagic acid”, “green extraction”, “conventional extraction”, “microwave-assisted extraction”, “ultrasound-assisted extraction”, “supercritical fluid extraction”, “microbial bioprocessing”, “fermentation”, “biotransformation”, “chemical characterization”, “standardization”, “antioxidant activity”, “anti-inflammatory activity”, “antibacterial activity”, “antifungal activity”, “antimicrobial resistance”, “multidrug-resistant pathogens”, “biofilm”, “antibiofilm activity”, “quorum sensing”, “mechanism of action”, “synergy”, “antibiotic adjuvant”, “prebiotic activity”, “gut microbiota”, “urolithins”, “food preservation”, “active packaging”, “animal nutrition”, “veterinary applications”, “plant pathogens”, “rhizosphere”, “agricultural applications”, “delivery systems”, “encapsulation”, “nanoformulations”, “scale-up”, and “circular bioeconomy”. These terms were used individually and in different Boolean combinations using the operators “AND” and “OR”.

The literature searches primarily focused on studies published between January 2000 and July 2026, with particular emphasis on research from the most recent 10–15 years. A limited number of earlier publications, dating back to 1993, were also included when they provided foundational contributions to the historical and traditional uses, phytochemical characterization, or biological properties of pomegranate. The methodological robustness of the selected studies was qualitatively assessed according to the clarity of plant-material identification, extraction and experimental conditions, chemical characterization, use of appropriate controls, suitability of the biological assays, replication, and reporting of quantitative outcomes. Given the heterogeneity of the evidence considered and the narrative design of the review, no formal risk-of-bias scoring system was applied.

Peer-reviewed original articles, reviews, and selected authoritative reports were included when relevant to botanical and agronomic variability, plant fractions and by-products, extraction technologies, phytochemical characterization, extract standardization, biological activities, microbial interactions, safety, formulations, or applications in food, biomedical, veterinary, agricultural, and environmental fields. Studies on crude extracts, enriched fractions, and purified compounds were considered when sufficient methodological information was provided. Preference was given to experimentally validated studies reporting clearly identified plant material, defined extraction conditions, chemical characterization, and appropriate biological assays. For antimicrobial studies, particular attention was paid to quantitative parameters such as minimum inhibitory, bactericidal, fungicidal, biofilm-inhibitory, and biofilm-eradicating concentrations, time–kill analyses, and combination indices. Non-peer-reviewed publications, conference abstracts without complete data, studies lacking methodological detail or experimental validation, duplicates, and articles not directly related to pomegranate-derived matrices or compounds were excluded. Titles and abstracts were screened first, followed by full-text assessment. Additional studies were identified from the reference lists of relevant articles and reviews. The selected literature was organized according to the main themes of the review.

## 3. Pomegranate as a Rich Source of Bioactive Compounds

### 3.1. Historical Evolution and Traditional Uses of Pomegranate

Pomegranate (*Punica granatum* L.) is an ancient fruit crop originating from Western and Central Asia and subsequently disseminated throughout the Mediterranean region [19,20,21,22,23]. Beyond its nutritional and cultural importance, different plant parts have long been used in traditional medicine, including for the management of infectious and gastrointestinal disorders. These ethnobotanical uses provided an early basis for subsequent investigations into the antimicrobial and microbiota-related properties of pomegranate-derived preparations.

Beyond its symbolic importance, pomegranate was incorporated into Ayurvedic, Persian, Mediterranean, and Middle Eastern medical practices. Fruits, peels, seeds, flowers, leaves, bark, and roots were traditionally used to manage gastrointestinal and inflammatory disorders, infections, and other ailments, as well as sources of natural antioxidant preparations [24,25]. Following its expansion from Western Asia and Europe to Africa, the Americas, and Australia, pomegranate became a globally cultivated crop, favored by its adaptability to arid and semi-arid environments. Domestication and artificial selection generated broad phenotypic diversification in fruit size and shape, aril coloration, acidity, sugar content, and phytochemical composition. Nevertheless, substantial genetic diversity persists among cultivars and local populations, reflecting distinct geographical origins and cultivation histories [26,27,28].

In recent decades, scientific interest has shifted from the ethnobotanical significance of pomegranate toward the genomic and phytochemical characterization of its bioactive constituents, with particular attention to polyphenols, flavonoids, and ellagitannins. This transition has repositioned *P. granatum* as a promising source of compounds with nutraceutical, pharmacological, and antimicrobial potential [29,30].

### 3.2. Botanical and Agronomic Characteristics of Punica granatum *L.*

*Punica granatum* L. is a perennial fruit-bearing species belonging to the family Lythraceae, native to the region extending from present-day Iran to northern India and currently cultivated throughout the Mediterranean basin, the Middle East, Asia, Africa, and the Americas [31,32,33,34,35,36,37]. Its adaptability to arid and semi-arid environments has contributed to its widespread cultivation and economic importance in Mediterranean, subtropical, and semi-arid regions [33,38]. Botanically, *P. granatum* grows as a shrub or small tree, while its characteristic fruit is a balausta with a thick pericarp enclosing numerous aril-covered seeds [39,40]. Besides the edible arils, peel, internal membranes, seeds, leaves, and other non-edible fractions represent relevant sources of bioactive phytochemicals, several of which have been associated with antimicrobial and antibiofilm properties [6,7,8,18,41,42,43,44,45,46,47,48].

More than 500 pomegranate cultivars have been described worldwide, showing marked differences in fruit characteristics and phytochemical composition [49,50]. This compositional variability is influenced by genetic background, geographical origin, ripening stage, pedoclimatic conditions, and cultivation practices [33,50,51,52,53,54,55]. Such variability is particularly relevant to the present review because differences in the abundance and profile of bioactive compounds may contribute to the heterogeneous antimicrobial responses reported for pomegranate-derived preparations.

### 3.3. Pomegranate Fractions and Agro-Industrial By-Products

During industrial processing, approximately 45–50% of pomegranate biomass is recovered as juice, while the remaining 50–55% consists mainly of peel, seeds, and internal membranes [47]. Peel is the predominant residue, representing approximately 40–50% of fruit weight, whereas seeds account for around 10–20%, although these proportions vary with cultivar and processing conditions [42,47,56,57]. These materials have traditionally been landfilled, incinerated, or composted, increasing disposal costs and potentially contributing to environmental pollution [58]. In addition to fruit-processing residues, cultivation and pruning generate leaves and other less-exploited plant fractions, including flowers, bark, and roots.

The edible arils are consumed fresh or processed into juice, concentrates, jams, syrups, wines, and functional beverages. Pomegranate juice is the main commercial product and contains sugars, organic acids, anthocyanins, flavonoids, and other polyphenols, whose concentrations vary according to cultivar, ripening stage, and processing method [59,60]. The expansion of juice production has consequently increased the availability of residues suitable for further valorization.

Among pomegranate residues, peel has received the greatest scientific and industrial attention because of its high content of hydrolysable tannins—particularly the α- and β-anomers of punicalagin, punicalin, gallagyldilactone, and pedunculagin—as well as ellagic acid derivatives, flavonoids, phenolic acids, pectin, and dietary fiber [18,61]. The high abundance of hydrolysable tannins and related phenolics also explains why peel is the pomegranate matrix most extensively investigated for antibacterial, antifungal, and antibiofilm applications. Its matrix comprises approximately 60% carbohydrates, 6–25% pectin, around 3% protein, and less than 1% lipids [13,56,62,63]. This composition supports the use of peel-derived ingredients in food preservation and active packaging, as well as in nutraceutical, cosmetic, and pharmaceutical formulations. Pomegranate seeds represent the second major processing residue and provide proteins, minerals, dietary fiber, and an oil yield generally ranging from 7.6% to 20% of seed weight, depending on cultivar, maturity, environmental conditions, and storage [41,47]. Although seed oil is increasingly exploited for nutraceutical and cosmetic applications, the defatted seed cake remains underutilized despite its content of proteins, polysaccharides, phenolic compounds, and dietary fiber.

Leaves, flowers, bark, and roots have been investigated less extensively than fruit-derived residues, although they contain tannins, flavonoids, phenolic acids, triterpenoids, and other specialized metabolites [64,65]. Leaves are particularly promising because approximately 150–200 kg may be generated for every tonne of harvested fruit during seasonal management and pruning [66]. Their abundance in hydrolysable tannins, flavonoids, and phenylethanoid glycosides makes them a renewable source of compounds for nutraceutical, pharmaceutical, cosmetic, and antimicrobial applications. Leaves are of particular microbiological interest because their polyphenol-rich composition has been associated with antimicrobial activity against both susceptible and antimicrobial-resistant microorganisms.

Overall, pomegranate residues are increasingly being regarded as inexpensive feedstocks for sustainable extraction and conversion into food, pharmaceutical, cosmetic, agricultural, and biomedical products. Their integrated valorization can reduce waste-management burdens, improve resource efficiency, and support circular biorefinery and zero-waste strategies, including the development of active and biodegradable packaging materials [58,67].

### 3.4. Phytochemical Diversity of Pomegranate-Derived Matrices

**Figure 2:**
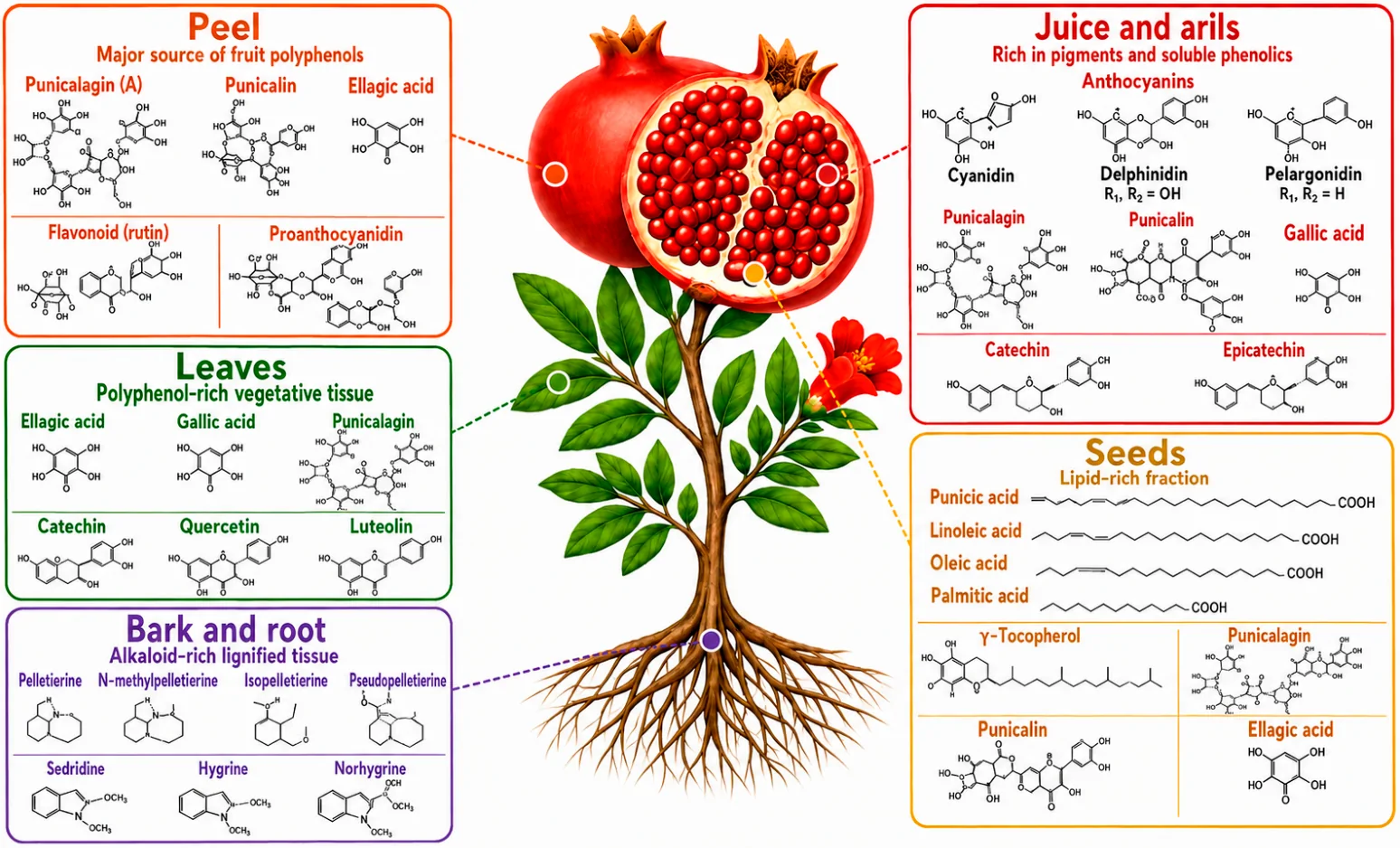
Matrix-specific phytochemical diversity of *Punica granatum* L. Schematic representation of the principal phytochemical classes associated with pomegranate peel, juice and arils, seeds, leaves, bark, and root.

Pomegranate (*Punica granatum* L.) is a chemically complex biological system characterized by marked phytochemical diversity among its anatomically distinct matrices. Extensive analytical investigations have identified a broad and heterogeneous spectrum of secondary metabolites whose qualitative and quantitative distribution differs among peel, juice and arils, seeds, leaves, and lignified tissues [68,69]. The principal matrix-specific phytochemical classes are summarized in Figure 2.

Pomegranate peel generally contains substantially higher concentrations of phenolic compounds than the pulp and is regarded as the fruit’s richest polyphenol source [48,70,71]. These metabolites derive mainly from the shikimate, pentose phosphate, and phenylpropanoid pathways and comprise tannins, flavonoids, and phenolic acids [72]. The tannin fraction includes ellagitannins, gallotannins, complex tannins, and condensed tannins [48,56,73,74,75]. Punicalagin, present as the α- and β-anomers, is the predominant ellagitannin and one of the most characteristic peel constituents [43,75,76,77,78]. Its hydrolysis yields ellagic acid, which also occurs in free and glycosylated forms [48]. Other reported tannins include granatin B, whereas rutin is classified as a flavonol glycoside rather than an ellagitannin [78,79]. Peel flavonoids include flavonols, anthocyanidins, and proanthocyanidins, with representative compounds such as catechin, quercetin, rutin, and luteolin [80,81,82]. Ellagic, gallic, chlorogenic, caffeic, ferulic, and cinnamic acids are among the main phenolic acids identified [56,69]. Peel is also rich in dietary fiber, primarily lignin, cellulose, uronic acids, xylose, arabinose, and galactose [48,83], while minor constituents include vitamins, steroids, mineral elements, and alkaloids such as acetylgrenadine derivatives, sedridine, pseudogrenadine, N-methylgrenadine, and isogrenadine [84,85].

Pomegranate juice and arils are particularly rich in hydrolysable tannins and anthocyanins but also contain flavonoids, lignans, organic acids, alkaloids, triterpenoids, phytosterols, and other minor constituents [75,86]. Their hydrolysable tannins comprise ellagitannins, gallotannins, gallagyl esters, and related phenolic acids. Punicalagin is the major ellagitannin, together with punicalin A and B, pedunculagin anomers, galloyl-HHDP hexoside, and galloyl-HHDP gluconate [2,59]. Additional galloylated compounds, including monogalloyl- and digalloyl-hexosides, and granatin B, have also been detected [3]. The phenolic acid fraction includes gallic, ellagic, caffeic, chlorogenic, *p*-coumaric, and ferulic acids and their derivatives [87]. Anthocyanins, which largely determine aril and juice colour, occur mainly as glycosylated derivatives of cyanidin, delphinidin, and pelargonidin [88,89]. Other flavonoids include dihydrochalcones, flavan-3-ols, flavonols, flavanones, and flavones, such as (+)-catechin, (−)-epicatechin, and (+)-gallocatechin [90]. Citric and L-malic acids are the predominant organic acids [91], while indolamines, lignans, triterpenoids, phytosterols, saponins, alkaloids, and vitamins, including vitamin C, occur at lower concentrations [92].

Pomegranate seeds are distinguished by their lipid fraction. Pomegranate seed oil is exceptionally rich in conjugated linolenic acids, particularly punicic acid, which generally represents 74–85% of total fatty acids; oleic, linoleic, and palmitic acids are also present [93,94]. The oil contains substantial amounts of tocopherols, with γ-tocopherol accounting for approximately 88–95% of the total tocopherol fraction [94]. Seed extracts also contain phenolic compounds, including punicalagin, punicalin, ellagic acid, gallic acid, and anthocyanin derivatives of cyanidin, delphinidin, and pelargonidin [89].

Pomegranate leaves exhibit a diverse profile dominated by polyphenols, with ellagic acid frequently reported as a major constituent [95]. Other compounds include gallic acid, catechin, epicatechin, quercetin, kaempferol, luteolin, apigenin, hydrolysable and condensed tannins, and anthocyanin derivatives [96,97]. The tannin fraction comprises granatins A and B, corilagin, strictinin, punicafolin, galloyl-glucose derivatives, brevifolin, brevifolin carboxylic acid, and punicalagin-related ellagitannins. Apigenin- and luteolin-derived glycosides, pyridinium alkaloids, and 2-methylpyran-4-one-3-*O*-β-D-glucopyranoside have also been identified [98]. In contrast, lignified tissues such as branches and twigs are characterized mainly by structural polyphenols [99]. Woody bark contains flavonols, flavanonols, flavones, flavanols, and tannins, including quercetin, kaempferol, myricetin, taxifolin, apigenin, luteolin, catechin, and epicatechin [100]. Bark and root bark also contain characteristic alkaloids: pelletierine, N-methylpelletierine, pseudopelletierine, sedridine, and norpseudopelletierine have been reported in bark, whereas hygrine and norhygrine occur mainly in root bark [101].

In addition to anatomical and genetic factors, harvesting procedures, mechanical transport, drying method, temperature, storage conditions, extraction solvent, solid-to-liquid ratio, extraction time, and analytical technique can substantially modify the recovered phytochemical profile [48,69,75,102]. Such methodological heterogeneity contributes to the differences reported in antioxidant, antibacterial, and anti-inflammatory activities. Comparability among studies therefore requires standardized procedures, detailed reporting of extraction and processing conditions, and chemical normalization using representative markers such as punicalagin [48,69,102].

## 4. From *Punica granatum* L. Biomass to Functional Extracts

### 4.1. Conventional Extraction Methods

Conventional extraction of bioactive compounds from pomegranate matrices mainly relies on solid–liquid techniques, including maceration, solvent extraction under agitation, Soxhlet extraction, infusion, and decoction. These methods are widely used because they are simple, inexpensive, and require limited equipment, although their efficiency depends on solvent polarity, extraction time and temperature, particle size, and solid-to-liquid ratio [13,103].

Water, methanol, ethanol, acetone, and their aqueous mixtures are commonly employed to recover phenolic compounds from pomegranate peel and other by-products. Methanol and acetone generally provide high recovery of total phenolics and ellagitannins, whereas aqueous ethanol represents a safer and more food-compatible alternative. However, differences in cultivar, drying, pretreatment, and extraction conditions limit comparisons among studies. Moreover, high extraction yield does not necessarily correspond to greater recovery of individual compounds such as punicalagin and ellagic acid [104]. Methanolic extraction has been reported to recover punicalagin, gallic and ellagic acids, rutin, and quercetin efficiently [105].

Soxhlet extraction remains a reference method for exhaustive extraction, particularly of pomegranate seed oil, but its high solvent consumption, prolonged processing, and elevated temperatures may promote the degradation of thermolabile compounds. Conventional techniques are therefore increasingly complemented by assisted extraction technologies that reduce time, energy demand, and solvent use. Nevertheless, they remain important benchmark methods for assessing the efficiency and sustainability of emerging processes [13,106].

### 4.2. Sustainable and Green Extraction Technologies

To overcome the limitations of conventional solvent extraction, several assisted and non-thermal technologies have been investigated for the sustainable recovery of bioactive compounds from pomegranate matrices. These approaches generally enhance cell disruption and mass transfer, allowing shorter processing times and reduced consumption of energy and organic solvents. Their combination with water, ethanol–water mixtures, and other food-grade solvents is particularly attractive for food, nutraceutical, and pharmaceutical applications. The operating principles, pomegranate matrices and target compounds, solvent systems, key process parameters, advantages, limitations, and representative studies of the main advanced and green extraction technologies are summarized in Table 1.

Ultrasound-assisted extraction (UAE) promotes acoustic cavitation, which disrupts plant tissues and facilitates solvent penetration. Its efficiency is mainly influenced by ultrasound power and frequency, treatment time, temperature, operating mode, particle size, solvent composition, and solid-to-liquid ratio. UAE has been successfully applied to pomegranate peel using water or aqueous ethanol, providing high recovery of punicalagin, ellagic acid, and other phenolic compounds while limiting solvent and energy requirements [107,108,109,110].

Microwave-assisted extraction (MAE), in contrast, relies on rapid volumetric heating generated by the interaction of microwaves with polar molecules. Microwave power, irradiation time, temperature, pressure, solvent dielectric properties, and solvent-to-solid ratio must be carefully controlled to maximize extraction while preventing the degradation of thermolabile phenolics. MAE can substantially shorten extraction times and has shown high efficiency in recovering phenolic compounds and punicalagins from pomegranate peel using water or aqueous ethanol [111,112].

Pressurized liquid extraction (PLE), including subcritical water extraction, maintains solvents in the liquid state at elevated temperature and pressure, thereby decreasing viscosity and surface tension and improving solvent penetration and solute diffusion. The main process variables include temperature, pressure, extraction time, solvent composition, flow rate, and number of extraction cycles. Water and ethanol are especially suitable because their polarity and selectivity can be modulated through temperature, enabling efficient recovery of phenolics with limited use of hazardous solvents. However, excessive temperatures may promote ellagitannin hydrolysis, phenolic degradation, and the formation of compounds such as 5-hydroxymethylfurfural. PLE using pressurized water–ethanol mixtures or natural deep eutectic solvents has been successfully applied to recover ellagic acid and α- and β-punicalagin from pomegranate peel [113,114].

High-pressure-assisted extraction (HPAE) enhances solvent penetration, cell-wall disruption, and membrane permeability through the application of hydrostatic pressure, generally without prolonged heating. Extraction performance depends on pressure level, treatment time, temperature, solvent composition, decompression conditions, and solid-to-liquid ratio. Studies performed at pressures of up to 600 MPa demonstrated that HPAE can improve the recovery of phenolics, tannins, flavonoids, anthocyanins, and punicalagin anomers from pomegranate peel while preserving antioxidant and antimicrobial properties [115,116]. Nevertheless, the high cost of pressure-resistant equipment and the complexity of industrial-scale operation remain important limitations.

Similarly, pulsed electric field-assisted extraction (PEF) induces electroporation of cell membranes, facilitating the release of intracellular compounds at relatively low temperatures. Electric-field strength, pulse width, pulse number, specific energy input, treatment time, matrix conductivity, and moisture content are the principal factors affecting extraction. PEF can be used as a pretreatment before aqueous or hydroalcoholic extraction and may selectively enhance the recovery of compounds such as ellagic acid from pomegranate peel [110]. However, its effectiveness is strongly matrix-dependent. Recent evidence showed that, despite extensive cell permeabilization, PEF produced only marginal improvements when applied before PLE to fresh pomegranate peel, in which the high initial phenolic concentration already generated a strong diffusional driving force [117].

Supercritical fluid extraction (SFE), particularly with supercritical CO_2_, represents a solvent-free or low-solvent approach for recovering non-polar and moderately polar compounds, including pomegranate seed oil, punicic acid, tocopherols, and phytosterols. Extraction selectivity depends on pressure, temperature, CO_2_ flow rate, extraction time, particle size, and the addition of polar co-solvents such as food-grade ethanol. Its main advantages include low-temperature operation, limited oxidation, and the absence of toxic solvent residues. Nevertheless, high investment costs and the low polarity of CO_2_ restrict the recovery of hydrophilic phenolics unless suitable modifiers or sequential extraction processes are employed [118,119,120].

Finally, enzyme-assisted extraction (EAE) uses pectinases, cellulases, hemicellulases, tannases, and commercial enzyme mixtures to hydrolyse cell-wall polysaccharides and release bound compounds. Its efficiency depends mainly on enzyme type and concentration, pH, temperature, incubation time, and particle size. Conducted predominantly in water under mild conditions, EAE can improve phenolic recovery while reducing energy and organic-solvent consumption. Enzymatic treatments with pectinase and cellulase have enhanced the recovery of phenolics and punicalagin from pomegranate peel, whereas cellulolytic enzymes combined with microwave or ultrasound treatment have provided further process intensification [115,121,122]. However, enzyme costs, treatment duration, enzyme inactivation, and process standardization remain relevant limitations.

Overall, these technologies offer more sustainable extraction while enabling the selective recovery and integrated valorization of phenolic, lipid, and polysaccharide fractions from pomegranate by-products [13,106].

**Table 1:** Comparative overview of advanced and green extraction technologies for the recovery of bioactive compounds from *Punica granatum* L. matrices.

Extraction Technology	Principle	Matrix/Target Compounds	Typical Conditions/Key Parameters	Key Advantages	Key Limitations	References
Ultrasound-assisted extraction (UAE) 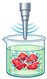	Acoustic cavitation enhances tissue disruption and mass transfer	Peel; punicalagins, ellagic acid, phenolics	Water/aqueous ethanol; power, frequency, time, temperature, solid-to-liquid ratio	Rapid; reduced solvent and energy use; efficient phenolic recovery	Excessive sonication may promote degradation; matrix- and equipment-dependent	[107,108,109,110,123]
Microwave-assisted extraction (MAE) 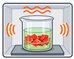	Rapid volumetric heating enhances cell disruption and solute diffusion	Peel; phenolics, punicalagins, ellagic acid derivatives	Water/aqueous ethanol; power, time, temperature, solvent polarity	Very rapid; high extraction efficiency; reduced solvent use	Risk of overheating; temperature control and scale-up challenges	[111,112]
Pressurized liquid extraction (PLE)/subcritical water extraction (SWE) 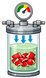	High temperature and pressure enhance solvent penetration and diffusion	Peel; ellagic acid, punicalagins, polar phenolics	Water/ethanol/deep eutectic solvents; temperature, pressure, flow rate, cycles	High efficiency; tunable selectivity; automation potential	Possible ellagitannin degradation; specialized equipment	[113,114]
High-pressure-assisted extraction (HPAE) 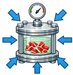	Hydrostatic pressure increases membrane permeability and solvent penetration	Peel; free and bound phenolics	Water/hydroalcoholic mixtures; pressure, time, temperature, decompression	Mild temperature; preservation of thermolabile compounds	High equipment and operating costs	[115,116]
Pulsed electric field-assisted extraction (PEF) 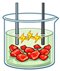	Electrical pulses induce membrane electroporation	Peel and moist by-products; phenolics	Aqueous/hydroalcoholic extraction; field strength, pulse conditions, conductivity, moisture	Low-temperature processing; short extraction time	Strong matrix dependence; specialized equipment	[110,117]
Supercritical fluid extraction (SFE) 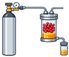	Supercritical CO_2_ selectively solubilizes lipophilic compounds	Seeds; seed oil, punicic acid, lipophilic compounds	Supercritical CO_2_ ± ethanol modifier; pressure, temperature, flow rate	Low solvent residues; limited oxidation; high selectivity	High investment cost; limited recovery of hydrophilic compounds	[118,119,120]
Enzyme-assisted extraction (EAE) 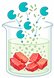	Cell-wall-degrading enzymes release bound compounds	Peel; free and cell-wall-associated phenolics	Water/buffers; enzyme type and dose, pH, temperature, time	Mild conditions; reduced solvent use; recovery of bound phenolics	Enzyme cost; longer treatment; condition-sensitive	[115,121,122]
Combined/sequential technologies 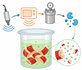	Integration of extraction mechanisms enhances cell disruption and mass transfer	Peel; broad phenolic fractions and ellagitannins	Food-grade solvents; technique-specific sequence and parameters	Potentially higher yield and selectivity	More complex optimization; higher cost; limited standardization	[13,106]

Abbreviations: CO_2_, carbon dioxide; EAE, enzyme-assisted extraction; HPAE, high-pressure-assisted extraction; MAE, microwave-assisted extraction; PEF, pulsed electric field-assisted extraction; PLE, pressurized liquid extraction; SFE, supercritical fluid extraction; SWE, subcritical water extraction; UAE, ultrasound-assisted extraction.

### 4.3. Integration of Extraction Processes and Prebiotic Activity

To maximize resource efficiency and direct pomegranate by-products toward higher-value applications, emerging biorefinery strategies increasingly combine green extraction and enzymatic pretreatment with microbial fermentation. Fermentation may be performed before extraction, as a biological matrix-disruption step, or after extraction, to selectively biotransform the recovered phytocomplex. Selected lactic acid bacteria, including *Lactiplantibacillus plantarum*, *Lacticaseibacillus paracasei*, and *Enterococcus faecium*, as well as yeasts such as *Saccharomyces cerevisiae* and filamentous fungi such as *Aspergillus niger*, can adapt to tannin-rich pomegranate matrices and modify their phenolic composition through strain-dependent hydrolytic activities. Microbial tannases, esterases, glycosidases, and other carbohydrate-active enzymes promote the cleavage of ester and glycosidic bonds, facilitating the conversion of complex ellagitannins, particularly punicalagin, into punicalin, ellagic acid, and other lower-molecular-weight derivatives. Indeed, different probiotic bacterial strains have been shown to transform punicalagin into ellagic acid, although the formation of urolithins was not detected, indicating that these more bioavailable metabolites generally require subsequent conversion by specialized members of the intestinal microbiota [124].

Fermentation can also operate as a pretreatment that enhances the subsequent recovery of bioactive compounds. Solid-state fermentation of pomegranate husks with *S. cerevisiae*, followed by ultrasound- and microwave-assisted hydroalcoholic extraction, produced markedly higher ellagic acid yields than either unfermented material or material fermented with *A. niger* [12]. Similarly, lactic fermentation with *L. plantarum* increased antioxidant activity and altered the abundance of ellagitannin derivatives in pomegranate juice, supporting the capacity of microbial metabolism to improve phenolic release and functional properties [125]. Importantly, microbial processing simultaneously affects the carbohydrate fraction. Recent evidence showed that *S. cerevisiae* fermentation promoted the depolymerization of native pomegranate peel pectin into fractions with molecular weights as low as approximately 26 kDa, while increasing tannin concentration in the recovered extracts and improving the utilization of the polysaccharide fraction by *Bifidobacterium breve* and *L. plantarum* [126].

Integrating extraction, enzymatic treatments, and microbial fermentation can generate multifunctional ingredients enriched in accessible phenolics and partially depolymerized prebiotic polysaccharides. This approach supports the development of synbiotic, probiotic, and postbiotic formulations while promoting pomegranate waste valorization. However, strain, fermentation conditions, and matrix composition must be standardized to ensure stability and reproducible functionality.

### 4.4. Chemical Characterization and Standardization of Extracts

Chemical characterization is essential for defining the composition, quality, authenticity, and reproducibility of pomegranate-derived extracts. Spectrophotometric assays, including total phenolic content (TPC), total flavonoid content (TFC), total anthocyanin content, and condensed tannin determination, are widely used for preliminary screening and for comparing extraction conditions [8,43,127,128]. When standardized reference compounds, analytical protocols, and clearly defined expression units are consistently used and reported, these assays can support meaningful comparisons among samples, extraction conditions, and studies. As class-level measurements, they provide rapid and accessible information that complements chromatographic and spectrometric techniques, which additionally enable the identification and quantification of individual phytochemical constituents. Accordingly, the combined use of these analytical approaches can provide a more comprehensive characterization of pomegranate-derived extracts. Compound-specific characterization can be obtained using HPLC- or UHPLC-based techniques coupled with diode-array detection and mass spectrometry, including HPLC-DAD, LC-ESI-MS/MS, UHPLC-QTOF-MS, and Orbitrap-MS. These approaches enable the identification and quantification of characteristic pomegranate constituents, including α- and β-punicalagin, punicalin, gallagic acid derivatives, ellagic and gallic acids, anthocyanins, flavonols, and other hydrolysable tannins [2,3,68,129]. HPLC analyses of pomegranate peel extracts obtained using ethanol, isopropanol, and hot or cold water have confirmed that solvent polarity and extraction temperature affect both the overall phenolic content and the recovery of individual compounds [61]. Similar variations have been observed among different fruit parts, cultivars, and extraction systems [16,130,131]. Chromatographic and mass-spectrometric methods can therefore support the selection of suitable chemical markers and the development of targeted extraction procedures for specific bioactive fractions. For quantitative standardization, analytical methods should preferably be validated in terms of selectivity, linearity, precision, accuracy, recovery, limits of detection and quantification, and analyte stability. Gas chromatography is mainly applied to the volatile, semi-volatile, and lipid fractions. Headspace solid-phase microextraction coupled with GC-MS can characterize volatile compounds and support aroma profiling or authenticity assessment, whereas the fatty acid composition of pomegranate seed oil is more commonly determined by GC-FID or GC-MS after conversion of fatty acids into their corresponding methyl esters. In this matrix, punicic acid represents the principal identity marker, while phytosterols, tocopherols, and minor fatty acids may provide additional information on oil quality and geographical origin [132]. FTIR spectroscopy provides rapid information on functional groups and can support spectral fingerprinting and comparative characterization of pomegranate-derived samples, although its limited molecular specificity generally requires confirmation by chromatographic methods. Nuclear magnetic resonance (NMR) provides complementary qualitative and quantitative information and has been successfully applied, alone or in combination with LC-MS, for comprehensive metabolite profiling of pomegranate extracts and juices [129,133]. The integration of these techniques with chromatographic and mass-spectrometric approaches can further strengthen the comparative characterization of pomegranate-derived samples. Untargeted LC-MS- and NMR-based metabolomics, combined with multivariate statistical methods such as principal component analysis, hierarchical clustering, partial least-squares discriminant analysis, and orthogonal PLS-DA, can further reveal compositional differences associated with the plant part, cultivar, geographical origin, maturity stage, agronomic conditions, processing, and extraction procedure. These approaches may also identify discriminatory metabolites for detecting adulteration or distinguishing authentic pomegranate products from mixtures containing cheaper fruit matrices [133,134,135]. Nevertheless, supervised classification models should be validated using independent sample sets to avoid overfitting and to confirm the robustness of the proposed chemical markers.

Comprehensive extract fingerprinting can complement class-level spectrophotometric measurements and single-marker quantification by providing a more detailed representation of the native phytochemical profile. Although standardization based on a limited number of analytical parameters or marker compounds can provide useful comparative information, it may not fully represent the complexity of the native phytochemical profile. Indeed, some commercial pomegranate products have been reported to contain low or undetectable amounts of characteristic ellagitannins, raising concerns regarding their compositional representativeness and equivalence [136]. Accordingly, a multi-marker strategy integrating spectrophotometric assessment, chromatographic fingerprints, and the quantification of representative compounds may provide a more comprehensive basis for compositional characterization and comparison. For peel-derived extracts, α- and β-punicalagin, punicalin, ellagic acid, and gallic acid may constitute a suitable marker panel, whereas juice standardization should additionally consider anthocyanins, organic acids, and sugars. Pomegranate seed oil requires a distinct marker profile based primarily on punicic acid, phytosterols, and tocopherols [135,137].

For comparative biological studies, reproducible characterization of pomegranate-derived preparations also benefits from adequate reporting of the main variables that may influence extract composition, including the botanical source and plant part, cultivar when available, pretreatment conditions, solvent composition, extraction parameters, extraction yield, and chemical composition. Concentrations should be expressed on a clearly defined dry-weight or dry-extract basis. Chemical fingerprints together with representative marker compounds may facilitate comparisons among pomegranate-derived extracts [137], particularly when evaluating antimicrobial, antibiofilm, and microbiota-modulating activities. Relevant stability and quality-control parameters should also be considered according to the intended application, particularly because pomegranate phenolics may undergo degradation during storage [138,139]. When extracts are intended for specific functional or antimicrobial applications, chemical standardization may also be complemented by an appropriate biological potency assay. Overall, sufficiently detailed and reproducible extraction, characterization, and analytical procedures facilitate meaningful comparison of biological activities across studies and support the reliable evaluation of pomegranate-derived ingredients.

## 5. Biological Activities of Pomegranate-Derived Compounds

### 5.1. Antioxidant and Anti-Inflammatory Activities

Pomegranate-derived extracts exhibit marked antioxidant activity, largely attributable to ellagitannins, particularly punicalagins, together with flavonoids, anthocyanins, and phenolic acids. In cell-free systems, these compounds can neutralize reactive oxygen and nitrogen species through electron- or hydrogen-atom transfer, chelate redox-active transition metals, and inhibit lipid peroxidation. Tannin activity is strongly influenced by the number and arrangement of phenolic hydroxyl groups and by galloyl, hexahydroxydiphenoyl, catechol-, and gallol-type moieties, which favour radical stabilization and metal coordination [140,141]. Flavonoid antioxidant capacity is likewise structure-dependent: an ortho-dihydroxylated B ring, the C2–C3 double bond conjugated with the 4-keto group, and a free 3-hydroxyl group generally enhance activity, whereas glycosylation often reduces direct radical-scavenging capacity [141,142]. In pomegranate extracts, antioxidant responses are strongly influenced by cultivar, plant part, matrix pretreatment, solvent polarity, and analytical method [48,56]. DPPH, ABTS, and FRAP assays rely on different reaction principles and therefore provide complementary information on antioxidant capacity; their combined use can contribute to a multifaceted characterization of pomegranate-derived preparations.

Beyond cell-free antioxidant activity, pomegranate polyphenols have shown antioxidant and anti-inflammatory effects in cellular and preclinical models through the modulation of multiple signalling pathways. In particular, punicalagin has been reported to activate Nrf2/Keap1 signalling and to modulate NF-κB, MAPKs, and PPARγ-related pathways, resulting in reduced oxidative stress and expression of pro-inflammatory mediators [143,144,145]. Pomegranate seed oil and its major conjugated fatty acid, punicic acid, have also shown antioxidant, anti-inflammatory, and tissue-protective effects in animal models of intestinal inflammation [146,147,148]. However, these findings remain predominantly preclinical, and differences in dose, formulation, route of administration, metabolism, and experimental models limit their direct extrapolation to humans.

Clinical evidence is more limited and heterogeneous. Studies conducted in different populations have reported reductions in selected inflammatory or oxidative-stress markers and improvements in specific clinical parameters, whereas other trials have shown no significant effects [149,150,151,152]. Differences in pomegranate matrix and extract composition, chemical standardization, dose, treatment duration, and study population further limit comparisons among studies. Therefore, although the available evidence supports the biological relevance of the antioxidant and anti-inflammatory properties of pomegranate-derived compounds, larger and better-standardized clinical studies are required to establish their translational significance. In the context of the present review, these activities provide complementary biological background to the subsequent discussion of antimicrobial and microbiota-related effects.

### 5.2. Antibacterial Activity

The increasing prevalence of antibiotic-resistant infections has stimulated interest in *Punica granatum* L. as a source of natural antimicrobial compounds. More specifically, pomegranate-derived preparations exhibit broad antibacterial activity against foodborne, environmental, and clinically relevant bacteria. However, their efficacy varies considerably according to the plant matrix, cultivar, extraction procedure, chemical composition, bacterial strain, and susceptibility-testing method. Representative antibacterial findings, including the most frequently investigated bacterial targets, pomegranate matrices, and quantitative outcomes, are summarized in Table 2.

Gram-positive bacteria generally show greater susceptibility to pomegranate-derived preparations than Gram-negative species. Peel, leaf, juice, and aril preparations have exhibited inhibitory or bactericidal activity against *Staphylococcus aureus*, *S. epidermidis*, *Enterococcus faecalis*, *E. faecium*, *Listeria monocytogenes*, *L. innocua*, *Micrococcus* spp., and several *Bacillus* species. Particularly marked responses have been reported for *L. monocytogenes*, with an inhibition zone of up to 58 mm following exposure to an aqueous peel extract, and for *S. aureus* and *B. cereus*, which showed MIC/MBC values of 5/10 mg/mL when treated with a hydroethanolic peel extract [45,153]. Notably, no significant cytotoxic effect was observed against normal MRC-5 fibroblasts, although some extracts showed antiproliferative activity against selected tumor cell lines [153]. High-pressure- and enzyme-assisted extraction also enhanced activity against *B. cereus*, *B. subtilis*, *S. aureus*, and *L. monocytogenes* compared with conventional aqueous extraction [115]. In the same study, high-pressure treatment at 300 MPa provided the highest total phenolic content, with punicalagin isomers among the most abundant phenolic constituents, whereas enzymatic treatment did not improve extraction yield. Phenolic content was also strongly associated with antimicrobial activity [115]. Nevertheless, substantial variability has been observed among preparations. Methanolic peel extracts from different cultivars displayed MIC values of 0.20–0.78 mg/mL, whereas the corresponding aqueous extracts generally required concentrations above 12.5 mg/mL [154]. Phenolic-rich juice extracts also inhibited *S. aureus*, *B. cereus*, *Micrococcus flavus*, and *L. monocytogenes*, with MIC and MBC ranges of 0.05–0.20 and 0.10–0.40 mg/mL, respectively [155]. Antibacterial activity has additionally been demonstrated in food systems: a commercial peel extract reduced *L. monocytogenes* growth by approximately 4.1 log CFU/g in refrigerated meat pâté, supporting its potential use in food preservation [156].

Although the lipopolysaccharide-rich outer membrane of Gram-negative bacteria generally limits their susceptibility, pomegranate preparations have shown activity against a broad range of enteric, foodborne, opportunistic, and environmental species. Investigated targets include *Escherichia coli*, including the enterohaemorrhagic O157 pathotype, *Salmonella enterica* serovars Typhimurium, Enteritidis, and Typhi, *Yersinia enterocolitica*, *Vibrio parahaemolyticus*, *Helicobacter pylori*, *Klebsiella pneumoniae*, *K. oxytoca*, *K. aerogenes*, *Acinetobacter baumannii*, *Enterobacter cloacae*, *Proteus mirabilis*, and several *Pseudomonas* species. A hydroethanolic peel extract showed MIC/MBC values of 20/40 mg/mL against *S. enterica* and 40/80 mg/mL against *E. coli*, consistent with the generally lower susceptibility of Gram-negative bacteria [45]. Similarly, aqueous and microwave-assisted peel preparations showed MIC values of 25 mg/mL against *E. coli* and *P. aeruginosa*, whereas methanolic extracts were frequently more active than aqueous ones [157,158]. Activity against *Pseudomonas* spp. is particularly noteworthy because this genus includes both opportunistic pathogens and important spoilage bacteria. An aqueous peel extract produced a 26-mm inhibition zone against *P. stutzeri*, while preparations tested against *P. putida* reduced viable counts by up to 3.15 log CFU/mL [158,159]. Responses nevertheless remain species- and preparation-dependent; for example, pomegranate juice inhibited *H. pylori* and *V. parahaemolyticus* but was ineffective against *Salmonella* spp. and *E. coli* O157 under the tested conditions [160]. Importantly, antibacterial activity is frequently concentration-dependent, and some preparations require relatively high extract concentrations to achieve inhibitory or bactericidal effects. For example, MIC/MBC values of 20/40 and 40/80 mg/mL were reported against *S. enterica* and *E. coli*, respectively [45], while MIC values of approximately 25 mg/mL were observed against selected Gram-negative bacteria [157]. Although these findings demonstrate biological activity, such concentrations may limit direct translation to systemic applications and emphasize the need to distinguish in vitro efficacy from practically achievable exposure levels.

Pomegranate extracts have also shown activity against clinically relevant resistant bacteria, particularly methicillin-resistant *S. aureus* (MRSA), resistant *Enterococcus* isolates, *K. pneumoniae*, *A. baumannii*, and multidrug-resistant *P. aeruginosa* [6,161,162]. Evidence concerning multidrug-resistant bacteria, biofilm-associated tolerance, and interactions with conventional antibiotics is discussed in greater detail in Section 5.4.

The antibacterial potency of pomegranate preparations is strongly influenced by the anatomical matrix. Peel is generally considered the most active source because of its high concentrations of ellagitannins, phenolic acids, and flavonoids. Leaves also represent a promising and renewable matrix, with some preparations exhibiting activities comparable to those obtained from peel [65,163]. By contrast, arils, juice, and seeds often show lower antibacterial potency and may require higher concentrations to inhibit less susceptible Gram-negative bacteria [160]. These observations support the preferential valorization of peels and leaves as sources of natural antibacterial ingredients.

Solvent composition and extraction technology further determine the qualitative and quantitative recovery of antibacterial compounds. Hydroalcoholic mixtures and polar organic solvents generally extract a broader spectrum of active phytochemicals than water alone, although aqueous preparations offer important advantages for food and biomedical applications [5,7,8,16,43,45,153,154,158,162,164]. Acidified solvents may additionally stabilize anthocyanins and modify the recovery of hydrolysable tannins, thereby affecting the antibacterial profile of the resulting extracts [165]. Advanced technologies, including ultrasound-, microwave-, high-pressure-, and enzyme-assisted extraction, can shorten processing time and improve the release of antibacterial phytochemicals. Nevertheless, their performance remains dependent on process optimization and chemical standardization [16,115,157].

Cultivar and bacterial background represent additional sources of variability. Cultivars such as “Hicaz nar”, “Gabsi”, and “Porphiroyeneti” differ in phenolic composition and antibacterial performance from widely cultivated varieties such as “Wonderful” [155,158,165]. Susceptibility may likewise differ among reference strains, clinical and veterinary isolates, foodborne bacteria, and phytopathogens such as *Xanthomonas campestris* and *Clavibacter michiganensis* [16,166].

**Table 2:** Representative antibacterial activities of *Punica granatum* L.-derived extracts and bioactive compounds against clinically and environmentally relevant bacteria.

Bacterial Targets	*P. granatum*-Derived Extracts or Bioactive Compounds	Main Outcomes	References
Gram-positive bacteria			
*Listeria monocytogenes*, *Staphylococcus aureus*, *S. epidermidis*, and *Bacillus* spp. (foodborne and spoilage-associated strains)	Aqueous, methanolic, butanolic, and hydroethanolic peel extracts	All extracts inhibited bacterial growth; the room temperature aqueous extract produced an inhibition zone of 58 mm against *L. monocytogenes*	[153]
*Bacillus cereus*, *B. subtilis*, MRSA, and *Listeria monocytogenes* (foodborne and clinically relevant strains, including an antibiotic-resistant phenotype)	Aqueous, high-pressure-, enzyme-, and combined enzyme–high-pressure-assisted peel extracts	High-pressure extracts were particularly active against *Bacillus* spp.; enzyme-assisted extracts showed lower MBCs against MRSA and *L. monocytogenes*	[115]
*Listeria monocytogenes* and *Staphylococcus aureus* (foodborne pathogenic strains)	Peel extracts obtained with different solvents; 80% methanol was the most active	Strong inhibition of both bacteria; activity was associated with the high phenolic content of the extract	[5]
*Enterococcus faecalis*, *Bacillus subtilis*, and *B. cereus* (foodborne, environmental, and opportunistic strains)	Ethanolic, methanolic, acidified alcoholic, acetone, and aqueous peel extracts	Acidified methanol and ethanol were the most active; inhibition zones: 17–36 mm	[165]
*Bacillus cereus* and *Staphylococcus aureus* (foodborne strains evaluated across cultivars and extraction methods)	Conventional and ultrasound-assisted peel and seed extracts	Inhibition zones up to 15.5 mm against *B. cereus* and 14.0 mm against *S. aureus*; activity varied with cultivar and extraction method	[16]
*Bacillus megaterium*, *Staphylococcus aureus*, *Corynebacterium xerosis*, *Enterococcus faecalis*, and *Micrococcus luteus* (human-associated and environmental strains)	Aril preparations from six pomegranate cultivars	Inhibition zones: 13–26 mm; MIC: 0.03 to >0.09 mg/mL; marked cultivar- and strain-dependent differences	[167]
*Bacillus subtilis* and *Staphylococcus aureus* (foodborne and clinically relevant strains evaluated across cultivars)	Methanolic and aqueous peel extracts from seven cultivars	Methanolic extracts showed MICs of 0.20–0.78 mg/mL; aqueous extracts generally showed MICs > 12.5 mg/mL	[154]
*Streptococcus mutans* ATCC 25175 and *Rothia dentocariosa* clinical isolate (oral reference and clinical strains)	Hydroalcoholic peel and juice extracts	Peel MIC/MBC: 10/15 mg/mL against both bacteria; juice extracts were less active	[46]
*Staphylococcus aureus* ATCC 25923 and *Bacillus cereus* ATCC 14579 (foodborne reference strains)	Hydroethanolic peel extract	MIC/MBC: 5/10 mg/mL; maximum inhibition zones: 21.0 and 20.5 mm, respectively	[45]
Clinical isolates of *Staphylococcus aureus*, including MSSA, MRSA, and PVL-positive community-associated MSSA (clinical isolates with different resistance and virulence profiles)	Aqueous rind extract, alone or combined with CuSO_4_	Extract MIC: 12.5–25 mg/mL; combination with CuSO_4_ reduced MICs and produced approximately 4-log reductions in viable counts	[161]
*Listeria monocytogenes*, *L. innocua*, and *Staphylococcus aureus* (foodborne pathogenic and indicator strains)	Pomegranate peel flour	MIC: 20–50 mg/mL; *S. aureus* was among the most susceptible species	[168]
*Listeria monocytogenes*, *Bacillus subtilis*, *B. cereus*, and *Staphylococcus aureus* (foodborne strains evaluated in a meat pâté model)	Commercial liquid pomegranate peel extract	Against *L. monocytogenes*, MBC: 24.7 mg dry extract/mL; approximately 4.1-log CFU/g reduction in refrigerated meat pâté	[156]
*Staphylococcus aureus*, *Bacillus cereus*, *Micrococcus flavus*, and *Listeria monocytogenes* (foodborne strains evaluated across juice cultivars)	Phenolic-rich juice extracts from “Persephone”, “Porphiroyeneti”, and “Wonderful” cultivars	MIC: 0.05–0.20 mg/mL; MBC: 0.10–0.40 mg/mL; “Porphiroyeneti” was generally the most active	[155]
*Bacillus subtilis* and *Staphylococcus aureus* (foodborne strains evaluated across sweet, sour–sweet, and sour cultivars)	Peel extracts from six sweet, sour–sweet, and sour cultivars	The PTO8 cultivar showed MICs of approximately 0.0015 mg/mL against *B. subtilis* and 0.0685 mg/mL against *S. aureus*	[169]
*Streptococcus mutans* ATCC 25175 and *Rothia dentocariosa* RD1 (cariogenic oral strains)	Hydroalcoholic peel extract	MIC: 10 mg/mL; MBC: 25 and 15 mg/mL, respectively	[8]
*Enterococcus faecium* and *Staphylococcus aureus*, including MRSA (clinically relevant Gram-positive ESKAPE strains)	Hydroalcoholic 50% peel extract	Concentration- and time-dependent antibacterial activity; intracellular bactericidal or bacteriostatic effects depending on the strain	[6]
Oral bacterial strains, including *Streptococcus mutans* and *S. sobrinus* (cariogenic oral strains)	Hydroalcoholic leaf extract	MIC: 0.00039–0.00625 mg/mL; bactericidal activity against most of the tested bacterial strains	[163]
*Staphylococcus aureus* ATCC 25923 and penicillin-resistant and methicillin-resistant *S. aureus* (reference and antibiotic-resistant strains)	Leaf extracts and fractions obtained with solvents of increasing polarity	Most active fraction MIC: 0.625–5 mg/mL; strain-dependent antibacterial activity	[65]
Clinical and veterinary isolates of *Staphylococcus aureus*, *S. pseudintermedius*, *Enterococcus faecalis*, *E. faecium*, and *Listeria monocytogenes* (human- and animal-associated isolates)	Hydroethanolic leaf, peel, and seed extracts	Leaf and peel extracts were more active than seed extracts; lowest MIC: 10 mg/mL	[162]
Gram-negative bacteria			
*Escherichia coli*, *Salmonella* Typhimurium, and *Pseudomonas aeruginosa* (foodborne and spoilage-associated strains)	Aqueous, methanolic, butanolic, and hydroethanolic peel extracts	Methanolic extract showed the strongest overall activity against Gram-negative bacteria	[153]
*Escherichia coli* ATCC 14169, *Pseudomonas aeruginosa* ATCC 27853, and *Proteus mirabilis* ATCC 29245 (clinically relevant reference strains)	Conventional and microwave-assisted aqueous, ethanolic, and acetone peel extracts	MIC: 25 mg/mL against *E. coli* and *P. aeruginosa*; ethanolic extracts showed MICs of 25 mg/mL against *P. mirabilis*	[157]
*Escherichia coli*, *Salmonella enterica*, and *Pseudomonas aeruginosa* (foodborne and clinically relevant strains)	Aqueous, high-pressure-, enzyme-, and combined enzyme–high-pressure-assisted peel extracts	High-pressure extraction improved activity against *P. aeruginosa*; effects against enteric bacteria were extract-dependent	[115]
*Escherichia coli*, *Yersinia enterocolitica*, and *Salmonella* Enteritidis (foodborne enteric pathogens)	Peel extracts obtained with different solvents; 80% methanol was the most active	Strong inhibition of *E. coli* and *Y. enterocolitica*; *S.* Enteritidis was less susceptible, with MIC of 4 mg/mL	[5]
*Escherichia coli* and *Klebsiella aerogenes* (enteric and opportunistic strains)	Ethanolic, methanolic, acidified alcoholic, acetone, and aqueous peel extracts	Acidified alcoholic extracts produced inhibition zones of 17–36 mm; aqueous extract was the least active	[165]
*Escherichia coli* and *Pseudomonas aeruginosa* (foodborne and opportunistic strains evaluated across cultivars and extraction methods)	Conventional and ultrasound-assisted peel and seed extracts	Inhibition zones up to 13 mm against *P. aeruginosa*; *E. coli* was resistant to the tested extracts	[16]
*Chromobacterium violaceum*; total coliforms and psychrophilic bacteria in alfalfa sprouts (quorum-sensing reporter strain and food-associated microbiota)	Aqueous and hydroethanolic peel extracts	Violacein production was reduced by approximately 43%; microbial counts decreased by 1.12–1.58 log CFU/100 g	[170]
*Pseudomonas stutzeri* CTSP36 (poultry meat-associated spoilage isolate)	Aqueous peel extract	Inhibition zone: 26 mm; 5% extract reduced cellular protein by approximately 98.5%	[159]
*Pseudomonas aeruginosa* and *Escherichia coli* (opportunistic and enteric strains evaluated across cultivars)	Aril preparations from six pomegranate cultivars	Inhibition zones: 13–26 mm; MIC: 0.03 to >0.09 mg/mL	[167]
*Escherichia coli* and *Xanthomonas campestris* (human-associated and phytopathogenic strains)	Aqueous leathery-exocarp extract	Marked bactericidal activity at 5–10 mg/mL; activity against *X. campestris* was comparable to the reference antibiotics	[166]
*Escherichia coli* and *Klebsiella pneumoniae* (enteric and clinically relevant strains evaluated across cultivars)	Methanolic and aqueous peel extracts from seven cultivars	Methanolic extracts showed MICs of 0.20–0.78 mg/mL; aqueous extracts generally showed MICs >12.5 mg/mL	[154]
*Salmonella enterica* ATCC 14028 and *Escherichia coli* ATCC 25922 (foodborne reference strains)	Hydroethanolic peel extract	MIC/MBC: 20/40 mg/mL against *S. enterica* and 40/80 mg/mL against *E. coli*	[45]
*Pseudomonas aeruginosa*, *Escherichia coli*, and *Salmonella* spp. (foodborne and spoilage-associated strains)	Pomegranate peel flour	MIC: 20–50 mg/mL; *Salmonella* spp. and *E. coli* were among the most susceptible bacteria	[168]
*Helicobacter pylori*, *Vibrio parahaemolyticus*, *Salmonella* spp., and *Escherichia coli* O157:H7 (gastrointestinal and foodborne pathogens)	Pomegranate juice	*H. pylori* and *V. parahaemolyticus* were susceptible; *Salmonella* spp. and *E. coli* O157:H7 were unaffected	[160]
*Pseudomonas putida* (food-spoilage-associated strain)	Aqueous and methanolic peel extracts	Aqueous extract reduced viable counts by up to 3.15 log CFU/mL; methanolic extract was more active	[158]
*Pseudomonas aeruginosa*, *Salmonella* Typhimurium, *Escherichia coli*, and *Enterobacter cloacae* (foodborne and opportunistic strains evaluated across juice cultivars)	Phenolic-rich juice extracts from three cultivars	MIC: 0.05–0.20 mg/mL; MBC: 0.10–0.40 mg/mL; cultivar-dependent activity	[155]
*Klebsiella pneumoniae*, *Acinetobacter baumannii*, *Pseudomonas aeruginosa*, and *Enterobacter* spp. (clinically relevant ESKAPE strains)	Hydroalcoholic 50% peel extract	Broad, concentration- and time-dependent activity against ESKAPE bacteria	[6]
*Salmonella* Typhi, *Pseudomonas aeruginosa*, and *Klebsiella pneumoniae* (enteric and opportunistic pathogens)	Peel extracts obtained with water, alcohols, ethyl acetate, or butanol	Methanolic extract MIC_50_: 7.5 mg/mL against *P. aeruginosa*; butanol and ethyl acetate extracts were inactive	[164]
*Escherichia coli* ATCC 25922 and penicillin-resistant and multidrug-resistant *E. coli* (reference and antibiotic-resistant strains)	Leaf extracts and fractions obtained with solvents of increasing polarity	Most active fraction MIC: 0.625–5 mg/mL; strain-dependent antibacterial activity	[65]
Clinical and veterinary isolates of *Escherichia coli*, *Klebsiella pneumoniae*, *K. oxytoca*, *Pseudomonas aeruginosa*, and *Salmonella* spp. (human- and animal-associated isolates)	Hydroethanolic leaf, peel, and seed extracts	Leaf and peel extracts were generally more active than seed extracts; lowest MIC: 10 mg/mL	[162]

Abbreviations: ATCC, American Type Culture Collection; CFU, colony-forming units; ESKAPE, *Enterococcus faecium*, *Staphylococcus aureus*, *Klebsiella pneumoniae*, *Acinetobacter baumannii*, *Pseudomonas aeruginosa*, and *Enterobacter* spp.; MBC, minimum bactericidal concentration; MIC, minimum inhibitory concentration; MIC_50_, concentration required to inhibit 50% of microbial growth; MRSA, methicillin-resistant *Staphylococcus aureus*; MSSA, methicillin-susceptible *Staphylococcus aureus*; PVL, *Panton*–*Valentine leukocidin*.

Pomegranate-derived compounds exert antibacterial effects through multiple and partially overlapping mechanisms, including disruption of the bacterial envelope, interference with cellular metabolism and communication, and modulation of virulence-associated processes. These molecular and cellular mechanisms are examined in detail in Section 5.5. Overall, chemically characterized preparations, standardized extraction protocols, and harmonized quantitative methods for determining MIC and MBC are required to compare antibacterial activity reliably across studies and support the development of reproducible pomegranate-derived antibacterial applications.

### 5.3. Antifungal Activity

The increasing incidence of opportunistic fungal infections, particularly those caused by drug-resistant pathogens, together with the limited availability of new antifungal drug classes, has stimulated the search for alternative strategies based on bioactive natural compounds [171,172,173,174]. In this context, *Punica granatum* L. has attracted considerable attention because of its complex phytochemical composition and reported activity against clinically relevant yeasts, dermatophytes, mycotoxigenic fungi, and post-harvest phytopathogens [4,14,44,175]. However, antifungal efficacy varies substantially according to cultivar, geographical origin, botanical part, extraction solvent, processing conditions, phytochemical composition, fungal species, and susceptibility-testing method. These differences limit direct comparisons among studies and highlight the need for chemically characterized and standardized preparations [18,176].

Among the principal fungal targets are *Candida* species, important opportunistic yeasts responsible for mucosal, bloodstream, and invasive infections. Although *C. albicans* remains the predominant species, non-*albicans Candida*, including *C. glabrata*, *C. tropicalis*, *C. parapsilosis*, *C. krusei*, and *C. dubliniensis*, have gained clinical relevance because of their variable susceptibility to conventional antifungal agents [171,173,177]. As summarized in Table 3, dichloromethane and methanolic pomegranate preparations inhibited several *Candida* species at MIC values of 0.0019–0.0039 mg/mL, whereas crude fruit extracts and their aqueous, ethyl acetate, and butanolic fractions showed MIC values of 3.9–15.6 mg/mL against reference strains of *C. albicans* and *C. parapsilosis* [178]. More recent studies using hydroethanolic peel extracts reported MIC and MFC values of 10–20 and 20–40 mg/mL, respectively, against clinical isolates of *C. albicans*, *C. glabrata*, and *C. parapsilosis* [44]. The hydroethanolic peel extract used in this study was chemically characterized, showing a TPC of 83.00 ± 0.52 mg GAE/g, with α-punicalagin, β-punicalagin, pedunculagin, and ellagic acid identified as major constituents [44]. The wide variation among reported concentrations partly reflects differences in extract composition, units, assay protocols, and fungal background, emphasizing the need for methodological harmonization. This variability also has translational implications. While some preparations or isolated compounds show antifungal effects at relatively low concentrations, others require concentrations in the mg/mL range. Therefore, apparent antifungal efficacy should be interpreted together with the administered concentration, extract composition, and intended route of application, rather than on the basis of growth inhibition alone. The emergence of multidrug-resistant *Candida auris* has further reinforced interest in alternative antifungal compounds. Evidence concerning complete pomegranate extracts against this species remains limited; however, ellagic acid has demonstrated inhibitory activity, supporting the potential use of selected pomegranate phenolics as lead compounds or antifungal adjuvants [179]. Pomegranate preparations have also been reported to interfere with *Candida* virulence-associated traits and persistent fungal communities. Since these effects extend beyond direct inhibition of planktonic growth, evidence concerning antifungal resistance and biofilm-associated tolerance is discussed separately in Section 5.4, while the underlying cellular and molecular mechanisms are examined in Section 5.5.

The antifungal spectrum of *P. granatum* also includes dermatophytes responsible for superficial infections of the skin, hair, and nails. Hydroalcoholic or hydroethanolic peel extracts inhibited reference strains and clinical isolates belonging to the *Trichophyton mentagrophytes* complex, *T. interdigitale*, and *T. rubrum*. As reported in Table 3, peel-extract MIC values generally ranged from 50 to 125 μg/mL, while isolated punicalagin showed MIC and MFC values of 62.5 and 125 μg/mL, respectively [175,180]. Activity was also retained against some clinical isolates showing reduced azole susceptibility. Moreover, combinations of pomegranate peel extract with ciclopirox olamine or zinc pyrithione produced synergistic or additive interactions, with FICI values ranging from 0.2 to 0.6, suggesting a possible role for pomegranate-derived compounds as topical antifungal adjuvants [180].

Beyond human fungal pathogens, pomegranate-derived preparations have shown activity against mycotoxigenic, food-spoilage, and phytopathogenic fungi. Aqueous, alcoholic, and organic-solvent peel extracts inhibited species belonging to *Aspergillus*, *Penicillium*, *Mucor*, *Alternaria*, *Botrytis*, *Botryosphaeria*, *Colletotrichum*, *Monilinia*, and *Rhizopus*, although susceptibility was strongly species- and extract-dependent [181,182,183,184]. Inhibition zones of approximately 10–23 mm have been reported against several *Aspergillus*, *Penicillium*, and *Mucor* species, whereas some *Fusarium* and *Rhizopus* strains were unaffected under the tested conditions [5,184]. Extracts obtained from peel, seeds, and leaves also reduced mycelial growth by approximately 28.46–42.77% and spore germination by 17.86–30.92% in post-harvest fungi such as *Botrytis cinerea*, *Penicillium italicum*, and *Rhizopus stolonifer* [181]. Particularly relevant results have been obtained against *Aspergillus flavus*, for which pomegranate peel extracts reduced aflatoxin B1 production by approximately 78.83–90.87%, indicating that their potential application may extend beyond growth inhibition to the control of mycotoxin contamination [185]. Pomegranate by-products have also shown promise in post-harvest protection. Methanolic and ethanolic peel extracts inhibited several fruit-associated pathogens, with ethanolic extracts and n-hexane fractions generally displaying the strongest activity [183]. In an ex vivo grape model, a concentrated peel extract completely prevented grey mould development caused by *B. cinerea* and retained preventive efficacy when applied up to 24 h before fungal inoculation [186].

Overall, the available evidence indicates that *P. granatum* possesses a broad but highly variable antifungal spectrum encompassing opportunistic yeasts, dermatophytes, mycotoxigenic moulds, and post-harvest phytopathogens. Peel represents the most frequently investigated and generally most active matrix, although leaf, seed, fruit, and juice-derived preparations may also contribute relevant antifungal compounds. Nevertheless, differences in fungal strains, extraction procedures, concentration units, and susceptibility endpoints currently hinder reliable comparisons. Chemically standardized extracts, harmonized MIC and MFC protocols, and validation in clinically or technologically relevant models are therefore required to support the development of reproducible antifungal, food-preservative, and crop-protection applications.

**Table 3:** Representative antifungal activities of *Punica granatum* L.-derived extracts and bioactive compounds against clinically and environmentally relevant fungi.

Fungal Targets	*P. granatum*-Derived Extracts or Bioactive Compounds	Main Outcomes	References
*Candida* spp.			
*Candida albicans* CBS-562, *C. dubliniensis* CBS-7987, *C. glabrata* B-07, *C. krusei* CBS-573, *C. parapsilosis* CBS-604, and *C. tropicalis* CBS-94 (opportunistic yeast strains; isolate sources not specified)	Dichloromethane and methanolic *P. granatum* extracts; botanical matrix not specified	MIC: 0.0019–0.0039 mg/mL; broad activity against *C. albicans* and non-*albicans Candida* species	[187]
*Candida albicans* ATCC 10231 and *C. parapsilosis* ATCC 22019 (reference strains associated with mucosal and invasive candidiasis)	Crude fruit extract and aqueous, ethyl acetate, and butanolic fruit-extract fractions	MIC: 3.9–15.6 mg/mL; ethyl acetate and butanolic fractions were among the most active	[178]
*Candida albicans* ATCC 90028 (reference strain used for fungal morphogenesis assays)	Methanolic pomegranate peel extract	MIC > 1 mg/mL; inhibition of germ-tube formation	[188]
*Candida albicans* y-EGFP reporter strain (reporter model for fungal growth and morphogenesis)	Pomegranate peel extract; extraction solvent not specified	>70% growth reduction; approximately 71.4% reduction in fungal load; inhibition of the yeast-to-hypha transition	[189]
*Candida albicans* UGPCA25, *C. glabrata* UGPCG25, and *C. parapsilosis* UGPCP25 (clinical isolates of opportunistic yeasts)	Hydroethanolic pomegranate peel extract	MIC: 10–20 mg/mL; MFC: 20–40 mg/mL; fungistatic and fungicidal activity; reduced germ-tube formation in *C. albicans*	[44]
Dermatophytes			
*Trichophyton mentagrophytes* ATCC 1481 and *T. rubrum* ATCC 28189 (reference dermatophyte strains associated with cutaneous infections)	Hydroalcoholic crude peel extract and isolated peel-derived punicalagin	Peel-extract MIC: 0.125 mg/mL; *T. rubrum* MFC: 0.25 mg/mL; punicalagin MIC/MFC: 0.0625/0.125 mg/mL; CGI: 0.0625 mg/mL	[175]
*Arthroderma* sp., *T. mentagrophytes* complex MTCC 24953, *T. mentagrophytes* MTCC 7687, and *T. rubrum* ATCC 28188 (reference dermatophyte strains associated with infections of the skin and cutaneous appendages)	Hydroethanolic pomegranate peel extract	MIC: 0.05–0.10 mg/mL; marked inhibition of fungal growth; dose-dependent intracellular leakage reported for *T. rubrum*	[180]
*T. mentagrophytes* var. *interdigitale* isolates DA-02, DA-07, DA-11, DA-12, DA-13, DA-17, DA-18, DA-19, DA20A, DA20B, and KA04 (cutaneous clinical isolates, including isolates with reduced azole susceptibility)	Hydroethanolic pomegranate peel extract, ethanol/water 1:1	MIC: 0.05–0.10 mg/mL; inhibition zones up to approximately 41 mm; activity retained against isolates with reduced azole susceptibility	[180]
*T. mentagrophytes* var. *interdigitale* KA04 and DA-19 and *T. rubrum* ATCC 28188 (cutaneous clinical and reference dermatophyte strains)	Hydroethanolic peel extract combined with ciclopirox olamine or zinc pyrithione	Synergistic or additive interactions; FICI: 0.2–0.6, with the strongest synergy generally observed for ciclopirox combinations	[180]
Mycotoxigenic and food-associated filamentous fungi			
*Aspergillus flavus* (corn and stored-maize mycotoxigenic isolates identified by ITS analysis)	Ethanolic, acetone, methanolic, and diethyl ether pomegranate peel extracts	Aflatoxin B1 reduction: 78.83–90.87%; modulation of genes involved in aflatoxin biosynthesis	[185]
*Aspergillus flavus* and *A. niger* (food-associated fungi; *A. niger* reported as a food isolate)	Aqueous, 80% methanol–water, and diethyl ether pomegranate peel extracts	Inhibition zones up to 10 mm against *A. flavus* and 12 mm against *A. niger*	[5]
*Aspergillus flavus*, *A. niger* ATCC 1015, *A. ochraceus*, *Mucor* sp., and *Penicillium chrysogenum* (mycotoxigenic and food-spoilage fungi; isolate origins otherwise not specified)	Aqueous, 70% ethanolic, methanolic, and DMSO pomegranate peel extracts	Inhibition zones: 10–23 mm; 70% ethanolic and DMSO extracts were generally among the most active	[184]
*Fusarium moniliforme* ATCC 38932, *F. oxysporum*, and *Rhizopus* spp. (mycotoxigenic or food-associated filamentous fungi)	Aqueous, 70% ethanolic, methanolic, and DMSO pomegranate peel extracts	No detectable antifungal activity under the tested conditions	[184]
*Aspergillus flavus* and *A. niger* (strawberry- and blueberry-associated isolates, respectively)	Methanolic and ethanolic pomegranate peel extracts and n-hexane, ethyl acetate, and aqueous fractions	Species-dependent inhibition; ethanolic extract and n-hexane fraction were generally the most active; *A. niger* showed comparatively lower susceptibility	[183]
Phytopathogenic and post-harvest fungi			
*Alternaria alternata* ACCC 36135, *Botryosphaeria dothidea* CAAS isolate, *Botrytis cinerea* CAAS isolate, *Colletotrichum gloeosporioides* CAAS strain, *Monilinia fructicola* ACCC 36262, and *Penicillium expansum* ACCC 30904 (post-harvest phytopathogenic strains)	Methanolic and ethanolic pomegranate peel extracts and n-hexane, ethyl acetate, and aqueous fractions	Broad but species-dependent inhibition; ethanolic extract and n-hexane fraction were generally the most active; *M. fructicola* was among the most susceptible fungi	[183]
*Rhizopus stolonifer* (post-harvest isolate obtained from decayed peaches)	Methanolic and ethanolic pomegranate peel extracts and n-hexane, ethyl acetate, and aqueous fractions	Significant inhibition; ethanolic extract was more active than the methanolic extract, and the n-hexane fraction showed the strongest activity	[183]
*Botrytis cinerea*, *Penicillium italicum*, and *Rhizopus stolonifer* (post-harvest isolates maintained at Ferdowsi University of Mashhad, Iran)	Aqueous and methanolic peel, seed, and leaf extracts from *P. granatum* cv. “Shishe Kab”	Mycelial-growth inhibition: 28.46–42.77%; spore-germination inhibition: 17.86–30.92%; methanolic preparations were generally more active	[181]
*Botrytis cinerea* (grape-associated post-harvest isolate)	Concentrated pomegranate peel extract	Complete inhibition of grey mould development on grapes; preventive efficacy when applied up to 24 h before fungal inoculation	[186]

Abbreviations: ACCC, Agricultural Culture Collection of China; ATCC, American Type Culture Collection; CAAS, Chinese Academy of Agricultural Sciences; CBS, Centraalbureau voor Schimmelcultures culture collection; CGI, conidial germination inhibitory concentration; DMSO, dimethyl sulfoxide; FICI, fractional inhibitory concentration index; ITS, internal transcribed spacer; MFC, minimum fungicidal concentration; MIC, minimum inhibitory concentration; MTCC, Microbial Type Culture Collection and Gene Bank; y-EGFP, yeast-enhanced green fluorescent protein.

### 5.4. Activity Against Multidrug-Resistant Pathogens and Microbial Biofilms

Antimicrobial resistance (AMR) is a major global health threat that progressively compromises the treatment of bacterial, fungal, viral, and parasitic infections. Multidrug-resistant (MDR) pathogens are particularly concerning because of their resistance to clinically relevant antimicrobial classes and their involvement in difficult-to-treat healthcare-associated infections [190]. Biofilm formation further increases persistence by limiting antimicrobial penetration and promoting metabolic heterogeneity, efflux activity, and persister-cell development [191]. Pomegranate extracts and by-products are therefore being investigated as complementary antimicrobial and antibiofilm agents, although they cannot yet replace conventional therapies (Table 4).

Activity has been reported against several MDR pathogens, including members of the ESKAPE group. Scaglione et al. [6] found that hydroalcoholic pomegranate peel extract inhibited reference and MDR isolates of *Staphylococcus aureus*, *Pseudomonas aeruginosa*, and *Acinetobacter baumannii*, with MICs of 4–12, approximately 10, and 20–30 mg/mL, respectively. MDR isolates were not consistently less susceptible than reference strains, indicating that antibiotic-resistance profiles do not necessarily predict sensitivity to pomegranate polyphenols. Activity against MRSA, vancomycin-resistant enterococci, MDR *A. baumannii*, resistant *S. aureus*, *S. epidermidis*, and *Escherichia coli* has also been reported, although *Klebsiella pneumoniae* generally showed lower susceptibility and marked strain-dependent variability was observed [161,162,180,192]. Activity has additionally been demonstrated against Panton–Valentine leukocidin-positive community-associated *S. aureus* isolates; however, PVL positivity is a virulence characteristic and should not itself be interpreted as evidence of multidrug resistance [161]. Although peel is the most investigated tissue, phenolic-rich leaf fractions have inhibited MRSA, penicillin-resistant *S. aureus*, and resistant *E. coli* isolates [65]. Epicatechin, kaempferol, and apigenin from leaf extracts were also identified as potential CTX-M-9 β-lactamase inhibitors through experimental and computational analyses [193]. Evidence outside clinical settings includes MDR *Salmonella* from dairy products, avian pathogenic *E. coli* O78 in broilers, and the phytopathogen *Ralstonia solanacearum* [194,195,196]. However, large comparative studies involving molecularly characterized clinical, food, veterinary, and environmental isolates remain scarce, particularly for carbapenemase-producing *K. pneumoniae* and *Enterobacter* spp. Susceptibility may depend on membrane permeability, efflux systems, surface composition, metabolic state, and resistance mechanisms, as well as cultivar, plant tissue, solvent, phenolic content, and extract stability [6,65]. An additional advantage of pomegranate-derived preparations is their potential use as antibiotic adjuvants. Checkerboard assays have identified synergistic or additive interactions between selected peel or leaf extracts and conventional antibiotics. These combinations reduced the concentrations required for agents such as amoxicillin, gentamicin, and cefotaxime against selected resistant *S. aureus*, *E. coli*, and *P. aeruginosa* isolates [7,65,157]. The proposed basis of this sensitizing activity includes increased membrane permeability, interference with energy-dependent efflux systems, and enhanced intracellular antibiotic accumulation. Nevertheless, the outcome remains dependent on extract composition, antimicrobial class, bacterial strain, and interpretation criteria; apparent synergy should therefore be confirmed using standardized checkerboard and time–kill assays.

**Table 4:** Representative activities of *Punica granatum* L.-derived extracts and bioactive compounds against multidrug-resistant bacterial pathogens and bacterial and fungal biofilms.

Microbial Target	*P. granatum*-Derived Extracts or Bioactive Compounds	Main Outcomes	References
Multidrug-resistant bacterial pathogens			
*Staphylococcus aureus*, *Pseudomonas aeruginosa*, and *Acinetobacter baumannii* (reference and MDR isolates; clinically relevant ESKAPE pathogens)	Hydroalcoholic pomegranate peel extract	MICs of 4–12 mg/mL against *S. aureus*, approximately 10 mg/mL against *P. aeruginosa*, and 20–30 mg/mL against *A. baumannii*. MDR isolates were not consistently less susceptible than reference strains	[6]
*Staphylococcus aureus* clinical isolates, including MRSA and PVL-positive community-associated strains (human clinical isolates with methicillin-resistant or virulence-associated phenotypes)	Aqueous pomegranate rind extract, tested alone or in combination with CuSO_4_	Activity against MSSA, MRSA, and PVL-positive community-associated isolates; extract MICs of 12.5–25 mg/mL. Combination with CuSO_4_ enhanced antibacterial activity and produced approximately 4-log reductions in viable counts	[161]
MRSA, vancomycin-resistant *Enterococcus* spp., MDR *Acinetobacter baumannii*, resistant *Staphylococcus aureus*, *S. epidermidis*, *Escherichia coli*, and *Klebsiella pneumoniae* (clinically relevant resistant isolates; exact strain composition varied among studies)	Pomegranate-derived preparations obtained from different botanical matrices	Broad but strain-dependent activity against resistant Gram-positive and Gram-negative bacteria; *K. pneumoniae* generally showed lower susceptibility	[162,192]
Resistant clinical and veterinary isolates of *Staphylococcus aureus*, *S. pseudintermedius*, *Enterococcus faecalis*, *E. faecium*, *Escherichia coli*, *Klebsiella pneumoniae*, *K. oxytoca*, *Pseudomonas aeruginosa*, and *Salmonella* spp. (human- and animal-associated isolates)	Hydroethanolic leaf, peel, and seed extracts	Leaf and peel extracts were generally more active than seed extracts; antibacterial activity was strongly strain-dependent, with the lowest reported MIC of 10 mg/mL	[162]
Penicillin-resistant and methicillin-resistant *Staphylococcus aureus* and resistant *Escherichia coli* (reference and antibiotic-resistant bacterial strains)	Pomegranate leaf extracts and fractions obtained using solvents of increasing polarity	The most active fraction showed MIC values of 0.625–5 mg/mL. Selected combinations with conventional antibiotics produced synergistic or additive effects	[65]
CTX-M-9 β-lactamase (ESBL-associated resistance determinant investigated experimentally and computationally)	Leaf-derived epicatechin, kaempferol, and apigenin	Compounds were identified as potential CTX-M-9 β-lactamase inhibitors; biochemical and microbiological confirmation remains necessary	[193]
MDR *Salmonella* spp. (isolates recovered from dairy products)	Pomegranate-derived preparation	Antibacterial activity reported against food-associated MDR *Salmonella* isolates	[194]
Avian pathogenic *Escherichia coli* O78 (veterinary isolate evaluated in broilers)	Pomegranate-derived preparation	Activity reported against an avian pathogenic *E. coli* strain in a veterinary context	[195]
*Ralstonia solanacearum* (resistant phytopathogenic bacterial strain)	Pomegranate-derived preparation	Growth-inhibitory activity reported against a plant-pathogenic bacterium, extending the evidence beyond clinical isolates	[196]
Resistant *Staphylococcus aureus*, *Escherichia coli*, and *Pseudomonas aeruginosa* isolates (antibiotic-resistant strains evaluated in combination assays)	Selected hydroalcoholic peel extracts, leaf-derived fractions, and aqueous, ethanolic, or acetone peel extracts combined with conventional antibiotics	Synergistic or additive interactions reduced the concentrations required for amoxicillin, gentamicin, and cefotaxime. Outcomes depended on extract composition, antibiotic, bacterial strain, and interpretation criteria	[65,157]
Bacterial and fungal biofilms			
*Staphylococcus aureus*, *Listeria monocytogenes*, *Salmonella* spp., and *Escherichia coli* (selected Gram-positive and Gram-negative strains forming monospecies biofilms)	Pomegranate-derived extracts	Biofilm reductions exceeding 70% in selected strains; Gram-negative bacteria were generally less susceptible, and some effects occurred at concentrations close to planktonic MICs	[197]
*Pseudomonas aeruginosa* (biofilm-forming opportunistic pathogen; strain details not specified in Section 5.4)	Pomegranate-derived extracts	Reduced biofilm formation, quorum-sensing signals, swarming motility, pyocyanin production, elastase, and protease activity	[198,199]
Gram-negative bacterial pathogens, including *Pseudomonas aeruginosa* (biofilm- and virulence-associated phenotypes; preliminary *Caenorhabditis elegans*–*P. aeruginosa* model)	Bioactive pomegranate-derived fraction	Inhibition of efflux-associated phenotypes, pigment production, motility, extracellular proteases, and biofilm development; preliminary protective activity in the *C. elegans*–*P. aeruginosa* model	[200]
*Pseudomonas aeruginosa* PAO1 (reference biofilm-forming strain; quorum-sensing model)	Pomegranate peel extract and purified punicalagin	Reduced biofilm formation and swarming motility; modulation of the las and rhl quorum-sensing systems	[201]
*Streptococcus mutans*, *S. oralis*, *S. mitis*, and *Rothia dentocariosa* (oral strains forming single- and multispecies biofilms)	Hydroalcoholic pomegranate peel extract	MBIC: 20–40 mg/mL; MBEC: 80–120 mg/mL. Higher concentrations were required to disrupt preformed biofilms than to prevent their formation	[8]
*Streptococcus sobrinus* (cariogenic oral strain; adhesion and established-biofilm models)	Hydroalcoholic pomegranate leaf extract	Adhesion reduced by up to 66.27% at twice the MIC; disruptive effects were also observed against established oral biofilms	[163]
*Candida albicans* ATCC 90028 (reference yeast strain; developing and preformed biofilms evaluated in vitro)	Methanolic pomegranate peel extract	BIC: 0.25 mg/mL; approximately 90% reduction of preformed biofilm biomass; inhibition of germ-tube formation	[188]
*Candida albicans* y-EGFP reporter strain (fluorescent reporter model of biofilm formation and fungal morphogenesis)	Pomegranate peel extract	Biofilm formation reduced by 51.3%; activity was detected at dilutions up to 1:16 or 1:32 depending on the analytical assay; inhibition of the yeast-to-hypha transition and modulation of tyrosol and farnesol production	[189]
*Candida albicans*, *C. glabrata*, and *C. parapsilosis* (clinical isolates forming developing and mature biofilms on polyvinyl chloride discs)	Hydroethanolic pomegranate peel extract	MBIC/MBEC values of 60/120, 20/40, and 20/60 mg/mL, respectively. Mature biofilm biomass was reduced by approximately 60%, 73%, and 62%, with decreased adhesion, matrix disruption, altered architecture, and cellular damage	[44]

Abbreviations: ATCC, American Type Culture Collection; BIC, biofilm inhibitory concentration; CTX-M-9, CTX-M-type extended-spectrum β-lactamase 9; ESKAPE, *Enterococcus faecium*, *Staphylococcus aureus*, *Klebsiella pneumoniae*, *Acinetobacter baumannii*, *Pseudomonas aeruginosa*, and *Enterobacter* spp.; ESBL, extended-spectrum β-lactamase; MBEC, minimum biofilm eradication concentration; MBIC, minimum biofilm inhibitory concentration; MDR, multidrug-resistant; MIC, minimum inhibitory concentration; MRSA, methicillin-resistant Staphylococcus aureus; MSSA, methicillin-susceptible *Staphylococcus aureus*; PVL, *Panton*–*Valentine leukocidin*; y-EGFP, yeast-enhanced green fluorescent protein.

Pomegranate extracts can also inhibit microbial adhesion, virulence, and biofilm development. Salim et al. [197] reported biofilm reductions exceeding 70% in selected *S. aureus*, *Listeria monocytogenes*, *Salmonella*, and *E. coli* strains, although Gram-negative bacteria were generally less susceptible and some effects occurred near planktonic MICs. Importantly, antimicrobial and antibiofilm activities were positively associated with the punicalagin, flavonoid, and chlorogenic acid content of the extracts, further highlighting the influence of chemical composition on biological efficacy [197]. Evidence obtained at sub-inhibitory concentrations more clearly supports specific anti-virulence activity. Pomegranate extracts reduced *P. aeruginosa* biofilm formation, quorum-sensing signals, swarming motility, pyocyanin production, elastase, and protease activity [198,199]. A bioactive fraction also inhibited efflux-associated phenotypes, pigment production, motility, extracellular proteases, and biofilm development in Gram-negative pathogens, with preliminary efficacy in a *Caenorhabditis elegans*–*P. aeruginosa* model [200]. Similarly, peel extract and punicalagin modulated the las and rhl quorum-sensing systems and reduced biofilm formation and swarming in *P. aeruginosa* PAO1 [201]. Antibiofilm activity has also been demonstrated against oral bacterial communities. A hydroalcoholic peel extract inhibited the formation of single- and multispecies biofilms produced by *Streptococcus mutans*, *S. oralis*, *S. mitis*, and *Rothia dentocariosa*, with MBIC values ranging from 20 to 40 mg/mL. Disruption of the corresponding preformed biofilms required higher concentrations, with MBEC values of 80–120 mg/mL [8]. A hydroalcoholic leaf extract similarly reduced *S. sobrinus* adhesion by up to 66.27% at twice the MIC and showed disruptive activity against established oral biofilms [163]. These results indicate that both peel- and leaf-derived preparations can affect initial attachment as well as the persistence of mature oral microbial communities. Mature biofilms are more difficult to eradicate, and MBEC values are generally higher than MBIC values. This relationship is also evident in fungal biofilm models. A methanolic pomegranate peel extract inhibited biofilm formation by *Candida albicans* ATCC 90028 at a biofilm inhibitory concentration of 250 μg/mL and reduced preformed biofilm biomass by approximately 90% [188]. In a fluorescent *C. albicans* reporter model, peel extract reduced biofilm formation by 51.3%, with activity detectable at dilutions up to 1:16 or 1:32 depending on the analytical assay [189]. Higher concentrations were required to disrupt mature oral mono- and multispecies biofilms than to prevent their formation [8]. In clinical *Candida albicans*, *C. glabrata*, and *C. parapsilosis* isolates grown on polyvinyl chloride discs, MBIC/MBEC values were 60/120, 20/40, and 20/60 mg/mL, respectively, with reduced adhesion, matrix disruption, altered architecture, and cellular damage [44]. The same treatment reduced mature biofilm biomass by approximately 60% for *C. albicans*, 73% for *C. glabrata*, and 62% for *C. parapsilosis*. Although this device-associated model is more representative than conventional microplate assays, the high concentrations required against mature biofilms remain a major translational limitation.

Overall, pomegranate-derived products show promising antimicrobial, antibiofilm, and anti-virulence activity against resistant microorganisms, but efficacy is often achieved at relatively high concentrations. A clear concentration–response relationship emerges from several studies: increasing extract concentrations generally enhances antimicrobial or antibiofilm effects, whereas disruption of established biofilms commonly requires substantially higher concentrations than inhibition of planktonic growth or initial biofilm formation. Conversely, modulation of virulence-associated traits and quorum-sensing pathways has in some cases been observed at sub-inhibitory concentrations, suggesting that biological effects are not restricted to direct microbial killing. Importantly, extract concentration alone does not fully explain bioactivity, since potency also depends on the abundance and relative proportions of active constituents, including punicalagin and other phenolic compounds. Accordingly, comparisons based solely on nominal extract concentrations should be interpreted cautiously when preparations differ in phytochemical composition and degree of standardization. Reproducibility is further limited by differences in cultivar, geographical origin, cultivation conditions, tissue, extraction technology, and phytochemical composition [16,197]. Since most evidence derives from controlled in vitro assays, future studies should prioritize standardized extracts, defined chemical markers, clinically achievable concentrations, and evaluations of toxicity, selectivity, stability, formulation, bioavailability, and in vivo efficacy in infection models, medical devices, food-contact surfaces, and complex veterinary or food matrices. The translational relevance of the concentration required should also be evaluated according to the intended application, since concentrations that are unrealistic for systemic administration may remain potentially applicable in topical formulations, oral-care products, food-contact systems, coatings, or surface treatments. At present, pomegranate products should be considered promising adjunctive, topical, or surface-based agents rather than validated substitutes for conventional antimicrobial therapy.

### 5.5. Mechanisms of Antimicrobial Action

**Figure 3:**
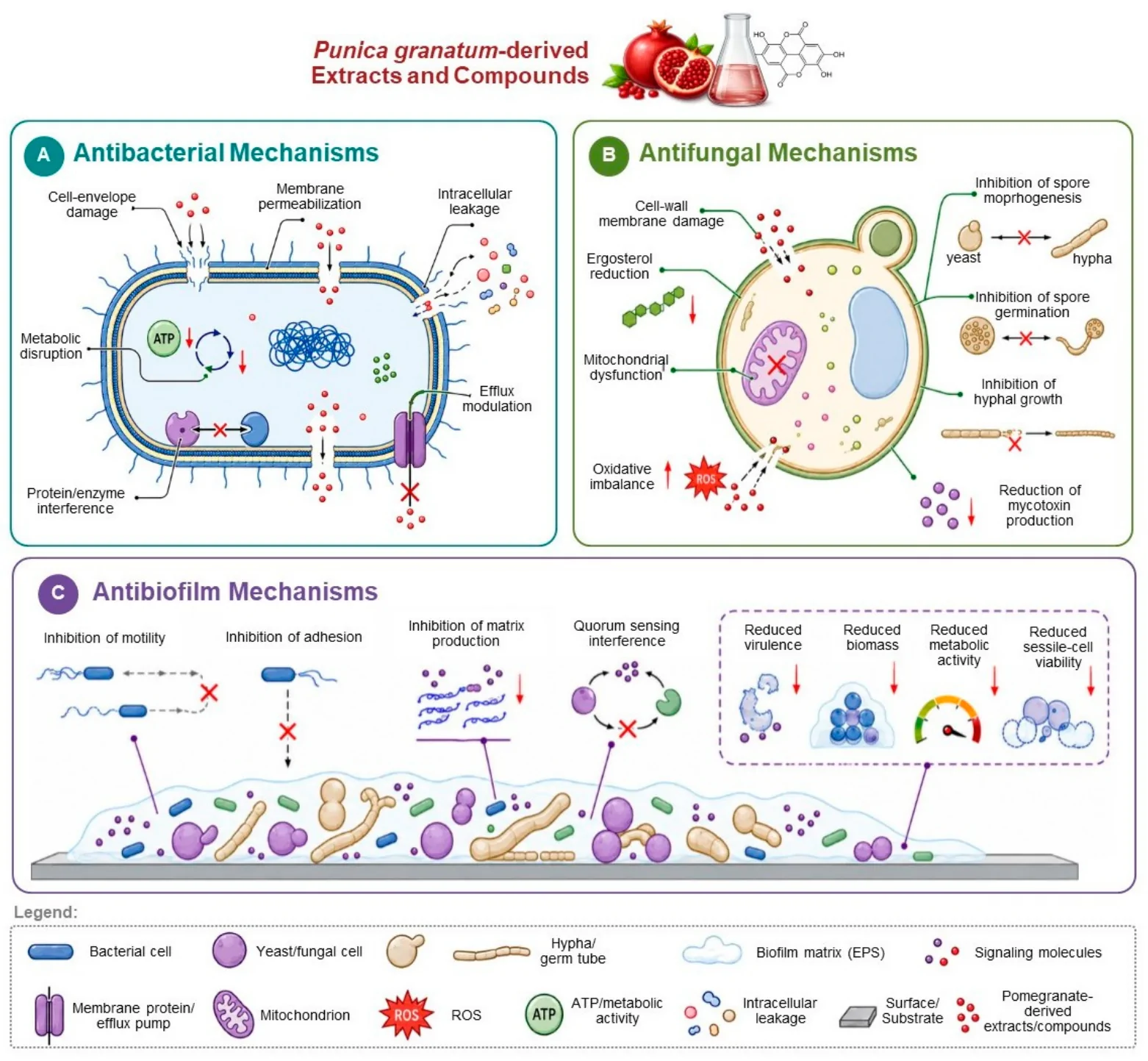
Main mechanisms underlying the antimicrobial activity of pomegranate-derived extracts and compounds. (**A**) Antibacterial mechanisms: cell-envelope damage, membrane permeabilization, intracellular leakage, metabolic disruption, efflux modulation and protein/enzyme interference. (**B**) Antifungal mechanisms: cell-wall and membrane damage, ergosterol reduction, mitochondrial and oxidative dysfunction, and inhibition of morphogenesis, spore germination, hyphal growth and mycotoxin production. (**C**) Antibiofilm mechanisms: inhibition of motility, adhesion, matrix production and quorum sensing, with reduced virulence, biomass, metabolic activity and sessile-cell viability.

The antimicrobial activity of pomegranate-derived extracts and compounds appears to result from the simultaneous perturbation of multiple microbial structures and physiological processes rather than from interaction with a single molecular target. The relative contribution of each mechanism depends on extract composition and concentration, as well as on the structural and metabolic characteristics of the target microorganism. The principal antibacterial, antifungal and antibiofilm mechanisms currently proposed are summarized in Figure 3.

Membrane and cell-envelope damage is the most consistently demonstrated antibacterial mechanism of pomegranate-derived compounds (Figure 3A). Punicalagin alters membrane potential and intracellular pH, increases permeability, promotes potassium and macromolecule leakage, and reduces intracellular ATP in *Salmonella Typhimurium* and *Vibrio parahaemolyticus* [202,203]. Similar effects, accompanied by ROS accumulation, metabolic inhibition, and reduced motility, were observed with fermented peel polyphenols against *Vibrio alginolyticus* [204]. Bioassay-guided fractionation identified α- and β-punicalagin as major antibacterial constituents against *Staphylococcus aureus* [205]. In MRSA, punicalagin also interacts with lipoteichoic acid and affects genes involved in peptidoglycan synthesis and teichoic-acid modification, causing cell-wall damage and ion leakage [206]. The lipopolysaccharide-rich outer membrane of Gram-negative bacteria may restrict the penetration of polyphenols and hydrolysable tannins, partly explaining their generally lower susceptibility compared with Gram-positive species [8,65,157]. Omics studies further indicate effects on fatty-acid and phospholipid biosynthesis, transport systems, iron acquisition, and central metabolism, supporting a multi-target response rather than a purely membranolytic action [207]. Specific resistance- and virulence-associated proteins may also be affected. Punicalagin inhibits sortase A-mediated anchoring of surface proteins and binds the SraP adhesin, thereby reducing adhesion, colonization, and β-lactam resistance-associated responses; these effects contributed to synergy with cefoperazone in MRSA models [208,209]. Increased intracellular norfloxacin accumulation and reduced efflux-associated phenotypes suggest modulation of efflux systems in MDR Gram-negative bacteria [200,210]. However, membrane depolarization itself can impair energy-dependent efflux, so direct pump inhibition still requires validation with transport assays and efflux-deficient mutants. The antibiotic-sensitizing activity observed against MDR Enterobacterales probably reflects combined effects on permeability, efflux, and metabolism [9]. Consistent with this interpretation, selected peel and leaf extracts decreased the concentrations of amoxicillin, gentamicin, and cefotaxime required against resistant *S. aureus*, *E. coli*, and *P. aeruginosa* isolates in checkerboard assays [7,65,157]. Protein binding and metal chelation may contribute further but remain less directly demonstrated. Hydrolysable tannins may interact with membrane-associated proteins and chelate divalent cations required for transport and cellular metabolism, although the relevance of these effects within whole extracts requires further experimental confirmation. Likewise, predicted interactions of leaf-derived epicatechin, kaempferol, and apigenin with CTX-M-9 β-lactamase require biochemical confirmation because docking alone does not establish enzyme inhibition [193]. Overall, direct disruption of the envelope and energy homeostasis is better supported than selective inhibition of DNA, RNA, or individual metabolic enzymes.

Antifungal activity also involves multiple surface and intracellular targets (Figure 3B). In *Candida* spp., peel extracts increase plasma-membrane permeability, induce structural damage, and promote intracellular leakage in planktonic and biofilm-associated cells [44,211]. Purified punicalagin causes cell-wall thickening, cytoplasmic disorganization, ergosterol depletion, cell-cycle arrest, and mitochondrial depolarization in *C. albicans* and *Cryptococcus gattii*. Since substantial ROS accumulation was not always detected, oxidative stress is not essential under all conditions [212]. Hydrolysable tannins can also inhibit fungal DNA topoisomerases I and II in vitro, although the contribution of this mechanism within complex extracts remains uncertain [213]. A hydroethanolic leaf extract similarly induced mitochondrial depolarization and enhanced fluconazole activity against *Cryptococcus* spp. [214]. Conversely, increased ROS contributed to the enhanced fungicidal and antibiofilm activity of pomegranate extract combined with Zn(II), indicating that oxidative damage may amplify membrane and mitochondrial dysfunction in specific formulations [215]. Although punicalagin and ellagic acid have been identified as relevant antifungal constituents, the activity of crude preparations generally reflects the combined action of hydrolysable tannins, ellagitannins, flavonoids, phenolic acids, and other components of the phytocomplex rather than the effect of a single metabolite [179,211]. Pomegranate products may also attenuate fungal virulence by reducing adhesion, germ-tube formation, yeast-to-hypha transition, and the release of the quorum-sensing molecules tyrosol and farnesol [44,189]. Predicted interactions with secreted aspartyl proteases remain preliminary and require direct enzymatic confirmation [211]. In dermatophytes, hydroalcoholic peel extracts and isolated punicalagin inhibited conidial germination and hyphal development. Increases in MIC in the presence of sorbitol additionally suggest that impairment of fungal cell-wall integrity contributes to the activity against *Trichophyton* spp. [175,180]. In filamentous fungi, peel extracts inhibit conidial germination and early hyphal development, reduce ergosterol, and can suppress mycotoxin production. In *Aspergillus flavus*, aflatoxin B1 reduction and downregulation of *aflR*, *aflC*, and *aflD* occurred even at concentrations with limited effects on biomass, suggesting partially growth-independent antimycotoxigenic activity. However, low sublethal doses occasionally stimulated toxin production, emphasizing the importance of dose-dependent stress responses [216]. A separate investigation reported modulation of the aflatoxin-biosynthetic genes *aflR*, *aflS*, *aflD*, *aflP*, and *aflQ*, together with marked reductions in aflatoxin B1 production, indicating that pomegranate peel extracts may interfere directly with fungal secondary metabolism and oxidative regulation [185]. Inhibition of mycelial growth, spore viability, and spoilage has also been observed against *Penicillium paneum*, *P. citrinum*, and *Aspergillus chevalieri* [217]. Current evidence supports effects on existing spores, germination, and early hyphal growth more strongly than inhibition of new conidium production.

Antibiofilm effects may result from sessile-cell killing or from interference with attachment, motility, extracellular polymeric substance production, signalling, and maturation (Figure 3C). Studies performed at sub-inhibitory concentrations are therefore essential to distinguish specific antibiofilm activity from general growth suppression. In *S. Typhimurium*, punicalagin reduced swimming, swarming, and the expression of flagellar, fimbrial, quorum-sensing, and virulence-associated genes [218]. In *S. aureus*, sub-MIC concentrations reduced surface hydrophobicity, biomass, metabolic activity, and biofilm compactness, while affecting planktonic growth only slightly [219]. Sortase A inhibition and reduced expression of adhesion-associated proteins may further impair surface colonization and cell–cell interactions [207,208]. Nevertheless, inhibition of initial attachment and matrix production is better demonstrated than active degradation of established staphylococcal biofilms. Quorum-sensing modulation is particularly relevant in *Pseudomonas aeruginosa*. Pomegranate peel extract altered autoinducer release and reduced biofilm formation independently of major effects on viability [198]. Peel extract and punicalagin also reduced swarming and biofilm development and downregulated the las and rhl systems, although direct binding of punicalagin to LasR remains based mainly on computational evidence [201]. Broader inhibition of QS-regulated pigments, proteases, motility, and biofilms has been reported in several Gram-negative pathogens, potentially involving both signalling and efflux modulation [200]. At sub-inhibitory concentrations, aqueous peel extract also reduced violacein production in *Chromobacterium violaceum*, supporting interference with quorum-sensing-regulated communication rather than a consequence of direct bacterial killing [170]. Similar changes in tyrosol and farnesol release indicate that signalling interference also occurs in fungal biofilms [189]. A methanolic peel extract additionally inhibited germ-tube formation and biofilm development in *C. albicans* ATCC 90028, supporting a relationship between altered morphogenesis and reduced biofilm establishment [188]. In oral bacterial models, a hydroalcoholic leaf extract reduced *Streptococcus sobrinus* adhesion by up to 66.27% at twice the MIC and disrupted established biofilms [163]. Against mature oral and *Candida* biofilms, pomegranate extracts reduced sessile-cell viability, adhesion, matrix organization, and three-dimensional architecture but generally required concentrations above those needed to prevent biofilm formation [8,44].

A final distinction should be made between complex extracts and purified compounds. Studies on punicalagin enable clearer target–effect relationships, improved dose standardization and more direct mechanistic interpretation. Conversely, peel and leaf extracts contain multiple constituents that may act additively, synergistically or antagonistically on membranes, oxidative balance, enzymes, transport systems and virulence pathways. This interpretation is consistent with evidence attributing antifungal activity to the collective action of tannins, ellagitannins, flavonoids, and phenolic acids rather than to a single dominant constituent [178,179,211,213]. This broader multi-target activity may reduce the likelihood that a single resistance mechanism abolishes antimicrobial efficacy, but it also complicates the identification of individual active compounds and contributes to variability among studies. Future investigations integrating membrane assays, metabolomics, transcriptomics, proteomics and targeted gene-validation approaches are therefore needed to distinguish primary molecular targets from secondary cellular responses.

### 5.6. Synergistic Interactions with Conventional Antimicrobials

The combination of pomegranate-derived extracts or purified compounds with conventional antimicrobials represents a promising strategy for enhancing drug activity against resistant microorganisms. Rather than replacing antibiotics or antifungals, pomegranate products may act as adjuvants that reduce effective drug concentrations, restore susceptibility or improve microbial killing. These interactions are commonly evaluated through checkerboard microdilution and the fractional inhibitory concentration index (FICI), with synergy generally assigned to FICI values ≤ 0.5, additivity to values > 0.5–1, indifference to values > 1–4 and antagonism to values > 4, although interpretative thresholds may vary. Time–kill assays provide complementary kinetic information, with synergy typically defined as a ≥2 log_10_ CFU/mL reduction by the combination compared with the most active single agent. Because checkerboard and time–kill results may not always agree, both methods should ideally be used.

Early studies showed that methanolic pomegranate peel extract potentiated chloramphenicol, gentamicin, ampicillin, tetracycline, and oxacillin against MRSA and methicillin-susceptible *Staphylococcus aureus*. The combination of 0.1× MIC extract with 0.5× MIC ampicillin was confirmed by time–kill analysis and prolonged the post-antibiotic effect [220]. An ellagic acid-rich preparation also enhanced moxifloxacin bactericidal activity against MRSA [221]. In resistant Gram-negative bacteria, pericarp extract reduced ciprofloxacin MICs and improved killing of fluoroquinolone-resistant, ESBL-producing *Escherichia coli* and *Klebsiella pneumoniae* and metallo-β-lactamase-producing *Pseudomonas aeruginosa* [222]. Reductions in antibiotic susceptibility and biofilm formation have also been reported in resistant *P. aeruginosa*, although some studies require confirmation through standardized kinetic assays [223]. Punicalagin has shown resistance-modifying activity with aminoglycosides, β-lactams, and fluoroquinolones against MDR *Salmonella enterica* serovars Typhi and Typhimurium and *E. coli*. At sub-inhibitory concentrations, it increased inhibition zones and reduced bacterial density in time–kill assays, although the magnitude of potentiation depended on strain, drug, and concentration [9]. Synergistic to borderline additive effects have also been reported for peel extract combined with azithromycin, with FICI values of approximately 0.12–0.53. Leaf fractions similarly reduced amoxicillin and cefotaxime MICs in selected resistant isolates [65]. Several complementary mechanisms may underlie these interactions. Membrane permeabilization may facilitate drug entry, whereas membrane depolarization can impair energy-dependent efflux and increase intracellular antibiotic retention. Increased norfloxacin accumulation in MDR *K. pneumoniae* and efflux-based screening in Gram-negative pathogens support functional modulation of efflux systems [200,210]. However, these findings do not necessarily demonstrate direct inhibition of specific transporters, because reduced membrane energy may indirectly impair efflux. Punicalagin also potentiated oxacillin against MRSA by decreasing *mecA* expression and PBP2a production [224]. Ellagic acid inhibited CTX-M-15 β-lactamase through a time-dependent interaction with the catalytic site, while ellagic and gallic acids altered antibiotic susceptibility in *S. aureus* strains expressing TetK and MepA pumps [225,226]. Thus, synergy may reflect combined effects on permeability, efflux, resistance enzymes, cell-wall functions, and microbial metabolism. Synergistic interactions have also been described against fungi. In *Candida* spp., pomegranate peel extracts, leaf fractions, and isolated constituents enhanced fluconazole activity and modulated virulence traits such as phospholipase production [178,227]. These effects may involve increased membrane damage, interference with ergosterol-dependent functions, or enhanced intracellular azole exposure but remain species- and strain-dependent. In *Cryptococcus* spp., a hydroethanolic leaf extract potentiated fluconazole and amphotericin B, possibly through mitochondrial dysfunction and impaired energy homeostasis [214]. Pomegranate peel extract also enhanced prochloraz activity against *Aspergillus* and *Fusarium*, delaying conidial germination and hyphal development. In *A. flavus*, the combination completely suppressed aflatoxin B1 production and downregulated *aflR*, *aflC*, and *aflD*, indicating effects on both growth and secondary metabolism [216]. Nevertheless, evidence remains limited for dermatophytes, *Penicillium*, Mucorales, and resistant non-*Candida* fungi.

Overall, pomegranate-derived products may enhance conventional antimicrobials by increasing permeability, impairing efflux and cellular homeostasis, and interfering with specific resistance determinants. These combinations could reduce required drug doses, but most evidence remains in vitro, based on limited strain panels and heterogeneous methods. Standardized checkerboard and time–kill assays, resistant clinical isolates, and appropriate in vivo models are needed, together with evaluation of pharmacokinetic compatibility, achievable tissue concentrations, stability, toxicity, and drug-metabolism interactions. Pomegranate–antimicrobial combinations should therefore still be regarded as experimental adjuvant strategies rather than clinically validated treatments.

### 5.7. Prebiotic Potential and Microbiota-Modulating Potential

Pomegranate-derived polyphenols have attracted increasing interest not only for their direct anti-inflammatory and antimicrobial properties, but also for their ability to interact with the intestinal microbial ecosystem and influence host physiology through microbiota-dependent pathways. The interaction between pomegranate-derived products and the intestinal microbiota is bidirectional. On the one hand, the gut microbiota is essential for converting poorly absorbed ellagitannins into more bioavailable metabolites; on the other, pomegranate polyphenols can influence microbial community structure and metabolic activity.

The first aspect of this interaction concerns the microbial biotransformation of ellagitannins. Punicalagins largely reach the colon, where they are hydrolysed to ellagic acid and progressively converted into intermediate urolithins, including M5, M6, D and C, and then into urolithin A, isourolithin A and urolithin B. After absorption, these metabolites circulate mainly as glucuronidated or sulfated conjugates [10,11]. Urolithin A has received particular attention for its anti-inflammatory, mitochondrial and metabolic effects, although pomegranate activity cannot be attributed exclusively to this metabolite, as unabsorbed phenolics, intermediate products and microbiota changes may also contribute [228]. Urolithin production varies markedly among individuals. Metabotype A subjects mainly produce urolithin A, metabotype B subjects additionally produce isourolithin A and/or urolithin B, whereas metabotype 0 individuals produce negligible amounts of final urolithins. These profiles reflect differences in microbial composition and may be influenced by age, health status, medication and long-term diet. Metabotype B has been associated more frequently with overweight, obesity and metabolic syndrome, while metabotype A appears more common in metabolically healthier subjects; however, these associations do not demonstrate causality [229,230]. Members of the Eggerthellaceae family contribute to this metabolism. *Gordonibacter urolithinfaciens* and *Gordonibacter pamelaeae* generate intermediate urolithins, whereas *Ellagibacter isourolithinifaciens* produces isourolithin A. Complete conversion probably depends on microbial consortia and cross-feeding rather than on a single universal producer, making the baseline microbiota a major determinant of the response to pomegranate polyphenols [231,232,233].

**Figure 4:**
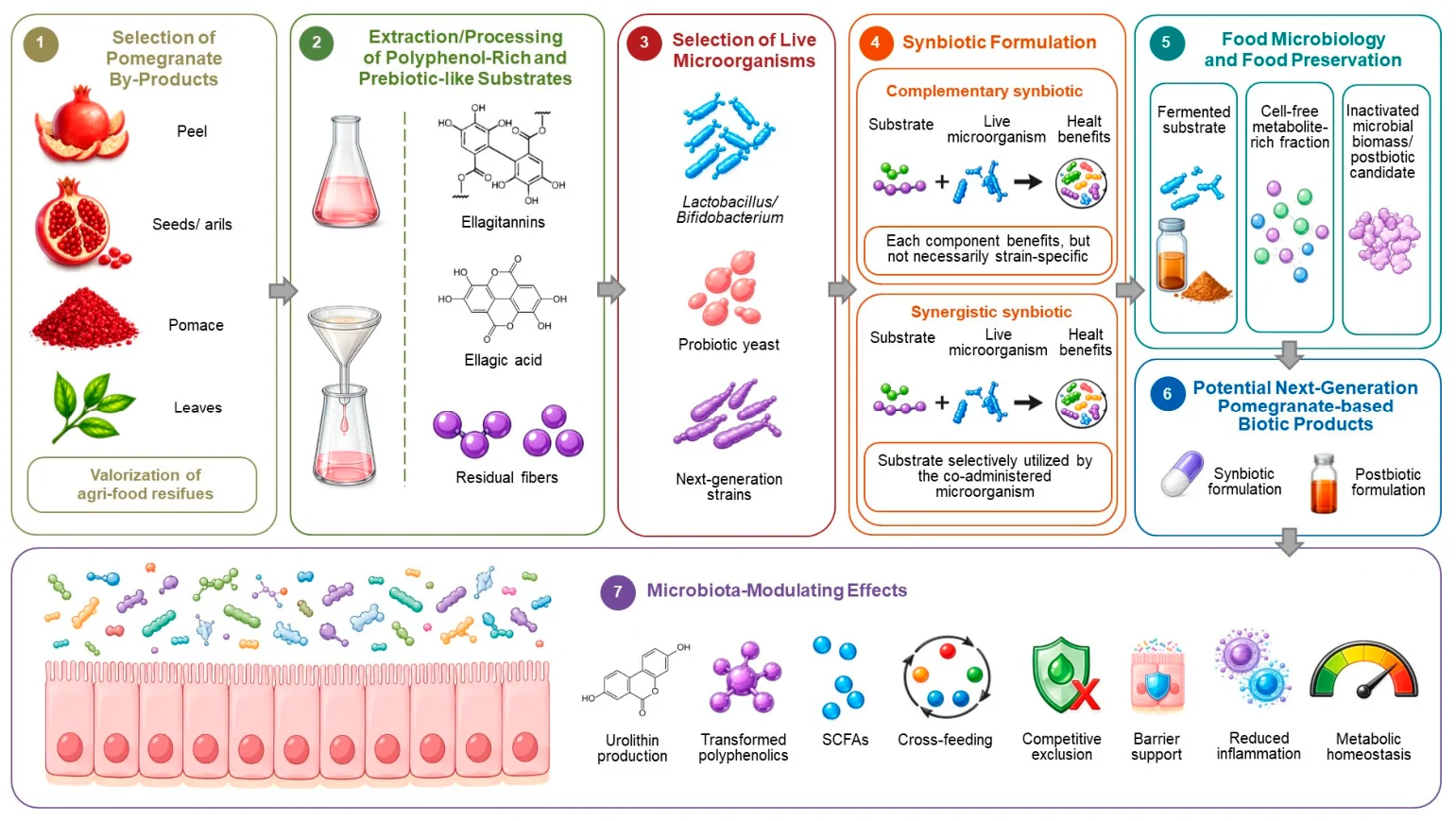
Conceptual workflow for the development of next-generation pomegranate-based biotic formulations.

The second aspect of the bidirectional interaction involves the ability of pomegranate polyphenols to modify microbiota composition and function. Experimental studies have reported increases in *Bifidobacterium*, *Lactobacillus*, *Akkermansia* and selected short-chain-fatty-acid-producing bacteria, together with reductions in Enterobacteriaceae, *Desulfovibrio* and other inflammation-associated taxa. These changes have been linked to improved barrier integrity, reduced endotoxaemia and attenuated inflammatory signalling, although genus-level variations should be interpreted cautiously because individual species and strains may have different functions. In healthy subjects, four weeks of pomegranate extract supplementation modified faecal microbiota and induced urolithin production, but responses differed between urolithin producers and non-producers [234]. Similarly, in vitro studies showed stimulation of selected bifidobacteria and lactobacilli and inhibition of enteric microorganisms [235]. However, these effects should not automatically be classified as prebiotic. A prebiotic must be selectively utilized by host microorganisms and confer a health benefit; microbial biotransformation or selective antimicrobial activity alone is insufficient. Pomegranate-mediated changes may instead result from substrate utilization, inhibition of susceptible microorganisms and cross-feeding through degradation intermediates. Sequencing data should therefore be complemented by growth kinetics, substrate-depletion analyses, isotope tracing and metabolomics. Functional changes may be as important as taxonomic shifts. Pomegranate supplementation has been associated with modulation of acetate, propionate and butyrate, which support epithelial metabolism, barrier integrity, immune regulation and metabolic homeostasis. However, responses are donor- and metabotype-dependent in fermentation models [236], while clinical findings remain inconsistent and are often based on small cohorts [237]. Pomegranate phenolics may also influence bile-acid, cholesterol and trimethylamine metabolism. In individuals with overweight, obesity or mild dyslipidaemia, responses were partly determined by urolithin-producing capacity and included changes in microbial pathways involved in bile-acid transformation and cholesterol homeostasis [238]. Reduced microbial trimethylamine production has also been suggested but remains primarily supported by preclinical evidence. These microbiota-dependent effects may contribute to improved intestinal permeability, inflammatory status and glucose or lipid homeostasis, although human evidence remains heterogeneous. In metabolic syndrome, concomitant treatments such as metformin may alter the baseline microbiota and obscure the independent effects of pomegranate supplementation [239]. Preliminary interventions in inflammatory bowel disease have also reported changes in barrier- and immune-related markers, but sustained clinical efficacy has not been established. Pomegranate-derived substrates may additionally be combined with selected microorganisms to develop microbiota-directed synbiotic formulations. Complementary synbiotics contain a beneficial microorganism and a substrate independently utilized by resident microbes, whereas synergistic synbiotics are designed so that the co-administered strain selectively utilizes the associated substrate. Demonstrating synergy therefore requires strain-specific substrate utilization or transformation, improved microbial persistence or activity, and a health benefit attributable to the complete formulation [240,241]. Peels, seeds, pomace and leaves may provide ellagitannins, ellagic acid and residual fibres suitable for this purpose. Simulated gastrointestinal digestion and colonic fermentation showed that pomegranate peel promoted butyrate and urolithin A production, increased *Bacteroides* and *Bifidobacterium*, and modified pathways related to carbohydrate, lipid and amino-acid metabolism [242]. Pomegranate juice also supported high viable counts of *Lactiplantibacillus plantarum* during fermentation and refrigerated storage, while an immobilized-strain beverage maintained viability above 8 log CFU/mL and improved phenolic and antioxidant characteristics [243]. These findings demonstrate technological compatibility but not necessarily selective utilization of the pomegranate substrate. Next-generation formulations should therefore combine chemically characterized substrates with well-defined lactobacilli, bifidobacteria, probiotic yeasts or emerging beneficial strains, as illustrated in Figure 4. Selection should consider not only microbial viability but also the production of urolithins, transformed phenolics and short-chain fatty acids.

Strain-dependent degradation of punicalagin into ellagic acid by probiotic bacteria demonstrates that microorganisms can actively transform pomegranate ellagitannins [124]. Nevertheless, a true synergistic formulation requires direct comparison of the complete combination with each individual component under gastrointestinally relevant conditions. Growth, viability, substrate depletion and metabolite production should subsequently be validated in complex microbiota models and target hosts using strain tracking, targeted metabolomics and integrated multi-omics approaches [244].

Fermentation may further support the sustainable valorization of pomegranate-processing residues by generating products with distinct compositions and functions. Fermentation of pomegranate juice with selected *L. plantarum* strains modified phenolic profiles and increased antioxidant activity, confirming that microbial metabolism can release or transform matrix constituents [125]. Depending on processing and fractionation, the resulting products may include a fermented substrate, viable microbial preparation, cell-free metabolite-rich fraction or inactivated microbial biomass; these categories should not be used interchangeably. A preparation containing viable microorganisms and a selectively utilized substrate may qualify as a synbiotic. By contrast, a postbiotic must contain inanimate microorganisms and/or their components and confer a demonstrated health benefit. A cell-free supernatant or fermented polyphenol extract should therefore not automatically be classified as a postbiotic [245]. Potential microbiota-directed effects include phenolic biotransformation, urolithin and short-chain-fatty-acid production, cross-feeding, competitive exclusion, barrier support and modulation of inflammatory and metabolic pathways. Animal studies also indicate that microbiota transfer from pomegranate-treated animals can reproduce part of the protective phenotype, supporting a causal contribution of microbiota modulation in inflammatory conditions [246]. Nevertheless, increased phenolic content, antioxidant activity or probiotic viability after fermentation does not demonstrate synbiotic synergy or postbiotic functionality. Appropriate controls should include the unfermented substrate, the microorganism cultured without the substrate, the complete fermented preparation, the cell-free fraction and the inactivated-cell fraction. Human evidence remains preliminary: a multispecies probiotic–pomegranate intervention increased diversity within *Bifidobacterium* and *Lactobacillus*, faecal butyrate and urinary urolithin A, including in subjects with limited baseline urolithin production. However, the use of multiple strains and a proprietary extract prevented attribution of the effects to individual components and confirmation of true strain–substrate synergy [247].

Overall, most available formulations remain complementary rather than mechanistically validated synergistic synbiotics. Future studies should use standardized pomegranate-derived substrates, defined microbial strains and factorial designs comparing individual components with the complete formulation. Selective substrate utilization, strain persistence, phenolic transformation, urolithin and short-chain-fatty-acid production, safety, and host outcomes should be assessed longitudinally, preferably with metabotype stratification and integrated multi-omics analyses. Such evidence is required to translate pomegranate by-products from promising fermentation substrates into validated next-generation synbiotic or postbiotic ingredients.

### 5.8. Other Biological Activities

Among the additional biological activities attributed to pomegranate, its extracts and phytochemicals have been investigated for their antiviral, antiparasitic, antitumor, cardiometabolic, neuroprotective, and hepatoprotective potential.

Several in vitro studies have reported antiviral effects against different viral pathogens, including influenza virus, herpes simplex virus type 1 (HSV-1), and Mayaro virus. These activities appear to involve multiple mechanisms, including interference with viral attachment and entry into host cells, direct virucidal effects, and inhibition of viral replication [248,249,250]. Similarly, antiparasitic properties have been described against protozoan and helminthic parasites, suggesting that pomegranate-derived compounds may impair parasite viability by disrupting cellular integrity and interfering with essential metabolic pathways [251,252]. A growing body of experimental evidence has also highlighted the antiproliferative and antitumor potential of *P. granatum* extracts and isolated phytochemicals, particularly punicalagin and ellagic acid. These effects have been associated with the modulation of oxidative stress, inflammatory signaling, apoptosis, cell proliferation, angiogenesis, and metastatic processes in different cancer models [253,254,255,256,257,258]. In addition, preclinical and emerging clinical evidence suggests potential cardiometabolic, neuroprotective, and hepatoprotective effects, largely related to the regulation of oxidative imbalance, chronic inflammation, lipid metabolism, and tissue damage [259,260,261,262,263,264,265,266]. Recent randomized clinical trials have further indicated beneficial effects of pomegranate supplementation on metabolic syndrome-related parameters, inflammatory biomarkers, osteoarthritis-associated inflammation, and skin health outcomes [267,268,269].

However, despite these promising findings, most available evidence still derives from in vitro and animal studies. Clinical validation therefore remains limited and requires standardized extracts, well-defined doses, and larger controlled trials before therapeutic applications can be established.

## 6. Applied and Biotechnological Potential of Pomegranate Extracts

**Figure 5:**
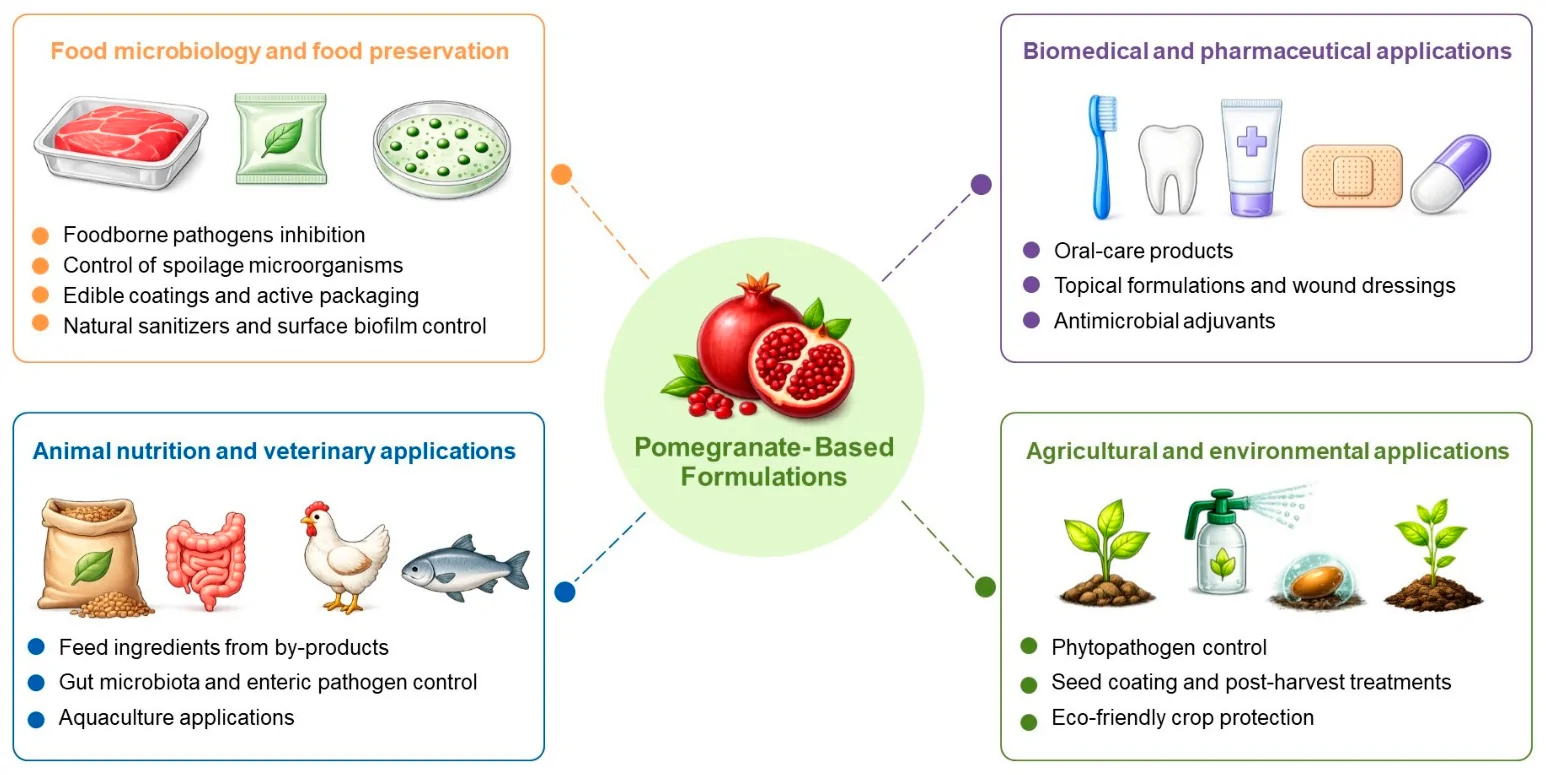
Main potential biotechnological applications of pomegranate-derived preparations.

Pomegranate extracts have potential applications in food, biomedical, veterinary, agricultural, and industrial sectors due to their antimicrobial, antioxidant, anti-inflammatory, and microbiota-modulating properties. They may be used in preservatives, packaging, feed, healthcare formulations, crop protection, and delivery systems. Their effectiveness depends on composition, dose, stability, bioavailability, safety, and matrix compatibility, while encapsulation and controlled-release technologies may improve functionality. As shown in Figure 5, these applications also support the sustainable valorization of pomegranate by-products within circular production models.

### 6.1. Applications in Food Microbiology and Food Preservation

Pomegranate-derived extracts, particularly those obtained from the peel, have been investigated as natural antimicrobial and antioxidant ingredients for controlling foodborne pathogens, spoilage microorganisms and oxidative deterioration. Their activity against bacteria, yeasts and filamentous fungi has encouraged both their direct addition to foods and their incorporation into edible coatings and active packaging systems. However, efficacy observed in culture media cannot be directly extrapolated to complex food products, because proteins, lipids and carbohydrates may interact with phenolic compounds, while pH, water activity and processing conditions may influence their stability and availability at microbial targets. Application studies conducted in real food matrices are therefore essential for assessing their technological potential. Encouraging results have been obtained in seafood products. In chilled bighead carp fillets, aqueous and ethanolic pomegranate peel extracts modified the spoilage microbiota, delayed quality deterioration and improved preservation during refrigerated storage [270]. In Pacific white shrimp, a chitosan coating containing pomegranate peel extract reduced melanosis and microbiological and biochemical deterioration while preserving sensory and textural quality during iced storage [271]. These findings support the potential use of pomegranate-derived preparations in seafood preservation, although their effectiveness depends on the extraction method, concentration, application system and characteristics of the treated product. Applications have also been investigated in meat and cereal-based products. The combination of pomegranate peel extract with vacuum packaging reduced lipid oxidation and microbial deterioration in refrigerated ground goat meat and cooked meat products [272]. More recently, pomegranate peel and olive leaf extracts improved the microbiological and oxidative stability of fresh meat, supporting the valorization of plant by-products as natural food additives [45]. In fresh filled pasta, the incorporation of pomegranate peel powder into the dough and filling slowed quality deterioration and extended refrigerated shelf life, demonstrating that pomegranate by-products may also be applied to flour-based foods [273]. Nevertheless, direct addition may produce changes in colour, texture, bitterness or astringency, and the concentration providing the greatest antimicrobial effect may not correspond to that associated with the highest sensory acceptability. Fresh and minimally processed fruits and vegetables represent another relevant field of application, because these commodities are particularly susceptible to water loss, tissue softening, enzymatic browning and fungal decay. Pre- and postharvest treatments with pomegranate peel extract reduced green mould development caused by *Penicillium digitatum* in citrus fruits, supporting its potential use in postharvest disease control [274]. Pomegranate peel extract has also been incorporated into chitosan–pullulan coatings for mangoes, where it contributed to reduced weight loss and improved retention of firmness, phenolic compounds and antioxidant activity during storage [275]. Similar dipping treatments have been investigated in date fruits, with reductions in yeast and mould counts and delayed quality deterioration without major adverse effects on sensory acceptability [192]. These findings indicate that pomegranate-derived preparations may contribute to the preservation of whole and fresh-cut produce, although their efficacy should be confirmed across different commodities, cultivars and storage conditions. Edible coatings and active packaging offer an additional strategy for limiting direct sensory interference while promoting the gradual release of bioactive compounds. Pomegranate peel extract has been incorporated into biodegradable films based on zein, chitosan, starch, gelatin and cellulose derivatives. Zein films containing the extract delayed microbial spoilage and oxidative deterioration in fresh cheese without markedly compromising sensory quality [276], whereas chitosan–starch films containing pomegranate peel extract and thyme essential oil prolonged the refrigerated shelf life of beef [277]. More recent carboxymethyl cellulose–gelatin films containing pomegranate peel extract have shown antioxidant, antibacterial and barrier properties relevant to meat preservation [278]. However, extract incorporation may also modify film colour, transparency, solubility, mechanical strength and water-vapour permeability, depending on concentration and interactions with the polymeric matrix. The antibiofilm activity reported against food-relevant microorganisms [197,279] also supports the potential use of pomegranate extracts in surface treatments and natural sanitizing systems. However, most evidence derives from planktonic cultures or static, single-species biofilm models rather than mature multispecies communities formed on food-contact materials. Comparisons among studies are further limited by differences in plant material, extraction methods, phenolic composition, dose, microbial targets and storage conditions, while the frequent use of multicomponent formulations makes it difficult to isolate the specific contribution of pomegranate extracts. Future studies should therefore use standardized preparations, appropriate controls and challenge tests in real food matrices and on industrially relevant surfaces. Antimicrobial stability, release from packaging, compatibility with sanitation procedures and possible sensory effects should also be evaluated before these extracts can be reliably applied as preservatives, active-packaging ingredients or natural sanitizing agents.

### 6.2. Biomedical and Pharmaceutical Applications

Pomegranate-derived preparations are increasingly being investigated in application-oriented biomedical formulations, particularly for oral care, wound management and gastrointestinal health. Among these areas, oral applications currently provide some of the most direct human evidence. Pomegranate extracts have been incorporated into mouthrinses and gels intended to control dental plaque, gingival inflammation and cariogenic microorganisms. A recent randomized clinical study found that a 30% pomegranate mouthrinse reduced plaque accumulation, gingival inflammation and oral bacterial counts over 21 days, with effects comparable to those of 0.12% chlorhexidine for several outcomes [280]. A 2024 comparative analysis similarly reported reductions in plaque, bleeding and gingival indices following the use of pomegranate-based mouthwashes or gels in patients with periodontal disease, supporting their possible use as adjunctive oral-care products rather than immediate substitutes for conventional antiseptics [281]. Nevertheless, differences in extract composition, concentration, treatment duration and comparator formulations currently limit direct comparison among clinical studies. Topical formulations and wound dressings represent another rapidly developing field. Pomegranate peel extracts have been incorporated into ointments, polymeric films, hydrogels and nanocomposite systems designed to provide local antimicrobial, antioxidant and anti-inflammatory activity while maintaining a moist environment and controlled release of bioactive compounds. In a recent applied study, a biodegradable hydrogel film based on carboxymethylated tapioca starch, β-cyclodextrin and pomegranate peel extract promoted wound closure in diabetic mice, while providing pH-dependent release and favourable biocompatibility properties [282]. Another recent study developed a hyaluronic-acid hydrogel containing pomegranate peel extract and silver nanoparticles for the management of *Candida albicans*-infected wounds. In vivo, the formulation reduced fungal burden, modulated inflammatory responses and improved tissue repair, although the contribution of the pomegranate fraction could not be fully separated from that of silver nanoparticles [283]. These studies demonstrate that pomegranate extracts are moving beyond simple topical testing towards multifunctional biomaterials evaluated in infected and metabolically impaired wound models. Gastrointestinal applications have also progressed from mechanistic and animal studies to preliminary controlled human interventions. In a randomized placebo-controlled trial involving patients with inflammatory bowel disease in clinical remission, 12 weeks of pomegranate juice consumption reduced faecal calprotectin and plasma endotoxin levels and modulated inflammatory gene-expression patterns [284]. In a separate prospective, randomized, double-blind, placebo-controlled study, oral supplementation with a standardized pomegranate extract was associated with changes in gut microbial composition, increased circulating urolithins, and higher concentrations of acetate and propionate, supporting its potential to influence microbiota-related metabolic activity in humans [237]. Nevertheless, both studies involved relatively small cohorts and should be regarded as preliminary evidence rather than confirmation of therapeutic efficacy. Combination with conventional antibiotics, antifungals or antiseptics remains an additional translational route, particularly for resistant microorganisms and biofilm-associated infections. Pomegranate-derived compounds may enhance antimicrobial activity through membrane damage, interference with adhesion and biofilm formation, or increased susceptibility to conventional agents. However, most combination studies remain laboratory-based, and synergy observed in checkerboard or biofilm assays does not automatically predict clinical benefit. In advanced hydrogels and nanocomposites, the simultaneous presence of silver, chitosan or other antimicrobial components also makes it difficult to determine whether the final activity is additive, synergistic or primarily attributable to the carrier material. Overall, the biomedical evidence now includes formulated products, animal wound models and a limited but growing number of human trials, rather than exclusively in vitro observations. However, the level of maturity differs markedly among applications: oral-care products have reached small randomized clinical studies, gastrointestinal formulations remain at an early proof-of-concept stage, and wound dressings are still predominantly supported by preclinical models. Further translation will require chemically standardized extracts, clearly defined doses, pharmacokinetic and bioavailability studies, validated release profiles and adequate assessment of local and systemic safety. Larger clinical trials should also distinguish adjunctive benefits from true therapeutic efficacy and determine whether pomegranate-based formulations offer reproducible advantages over established antiseptics, dressings or anti-inflammatory treatments.

### 6.3. Applications in Animal Nutrition

Pomegranate by-products, particularly peels, seed pulp, pomace and, to a lesser extent, leaves, have been investigated as functional feed materials because they provide fibre, residual nutrients and phenolic compounds with antioxidant and antimicrobial properties. Their inclusion in animal diets may contribute to the valorization of agricultural residues while supporting intestinal health, immune function and resistance to enteric pathogens. In broilers challenged with avian pathogenic *Escherichia coli*, supplementation with fermented or unfermented pomegranate peel influenced growth performance, intestinal morphology and cecal microbiota, with fermentation generally enhancing the functional value of the material [195]. In ruminants, pomegranate residues have mainly been evaluated as alternative feed ingredients and sources of tannins. Pomegranate seed pulp was incorporated into diets for growing Awassi lambs without adverse effects on animal health or meat quality, supporting its potential as a partial substitute for conventional feed components [285]. In dairy cows, graded dietary inclusion of pomegranate peel was assessed for its effects on intake, milk production, methane and nitrogen losses and metabolic health indicators. Although methane production was not significantly reduced, the study demonstrated that the peel could be included at controlled levels without major detrimental effects, while confirming the importance of dose-dependent evaluation [286]. Pomegranate leaves represent a less explored but potentially useful biomass for ruminant nutrition. Their practical development has focused primarily on ensiling rather than direct supplementation in livestock. Fermentation of pomegranate leaves with yogurt-derived lactic acid bacteria and molasses improved several silage fermentation and nutritional characteristics, suggesting a possible route for converting seasonal leaf residues into preserved forage materials [287]. However, this evidence derives mainly from silage-quality and in vitro ruminal evaluations; controlled feeding trials are still required to establish palatability, digestibility, animal performance and the effects of leaf tannins under farm conditions. Aquaculture is another promising area of application. Dietary pomegranate peel extract improved growth and haemato-immunological responses in rohu fingerlings challenged with *Aeromonas hydrophila*, with the intermediate inclusion level producing better outcomes than higher concentrations [288]. Similar supplementation enhanced selected health and immune parameters in common carp challenged with *Aeromonas veronii* [289]. These results support the use of pomegranate-derived additives as complementary tools for health management and possibly for reducing routine dependence on prophylactic antimicrobials, although they do not demonstrate that phytogenic supplements can replace therapeutic treatment during established infections. Overall, current evidence is based mainly on controlled feeding trials and experimental challenge models. Broader application requires standardized characterization of the plant material, dose–response studies, assessment of feed stability and palatability, and longer field trials across different species, production systems and environmental conditions. Potential antinutritional effects of tannins, residues in animal-derived foods, environmental consequences and cost-effectiveness should also be considered before pomegranate by-products, including leaf-derived preparations, can be widely adopted as functional feed ingredients.

### 6.4. Agricultural and Environmental Applications

Pomegranate-derived extracts and processing residues have attracted increasing interest as natural crop-protection agents and components of more sustainable agricultural systems. Polyphenol-rich peel extracts have demonstrated antibacterial and antifungal activity against several economically relevant phytopathogens, although most available evidence still derives from in vitro assays or experiments conducted under controlled conditions. More application-oriented studies have nevertheless provided encouraging results. Pomegranate peel extract applied as a foliar spray or seed coating reduced black rot symptoms and seed-borne infection caused by *Xanthomonas campestris* pv. *campestris* in *Brassica rapa*. At the concentrations tested, the treatments did not adversely affect seed germination or plant health, while the higher doses produced control levels comparable to those obtained using conventional copper- or heat-based treatments [290]. These findings support the development of pomegranate-derived seed treatments and botanical bactericides, although their performance should be confirmed across different crop varieties, seed lots and environmental conditions. Applications against fungal diseases have also been investigated. Field treatments with pomegranate peel extract during olive flowering reduced flower colonization and the subsequent severity of anthracnose caused by *Colletotrichum* spp. and olive leaf spot caused by *Venturia oleaginea*, suggesting a potential role in preventive crop-protection programmes [291]. Extracts obtained from pomegranate peel and seeds have additionally inhibited soil-borne and foliar pathogens, although their efficacy varied substantially according to the plant fraction, extraction solvent, concentration and target microorganism [292]. More recently, pomegranate peel extracts and individual polyphenols showed broad antifungal activity against fungi associated with postharvest fruit decay, including *Colletotrichum gloeosporioides*, *Rhizopus stolonifer*, *Aspergillus niger*, *Alternaria alternata*, *Monilinia fructicola* and *Penicillium expansum* [183]. Such preparations could potentially be incorporated into natural coatings, dipping solutions, sanitizing treatments or other postharvest preservation systems. However, most of these results were obtained under laboratory conditions, and activity in artificial media may not directly translate to complex fruit surfaces, storage environments or commercial supply chains. Further translational approaches include the treatment of perennial crops and systemic vascular infections. Endotherapeutic administration of pomegranate peel extract has been investigated in olive trees affected by *Xylella fastidiosa* subsp. *pauca*, suggesting that trunk-delivery systems may facilitate the local distribution of antibacterial phytochemicals within woody tissues [293]. Nevertheless, the effectiveness of such approaches may be influenced by extract stability, xylem mobility, tree physiology, disease stage and repeated-treatment requirements. More generally, evidence from replicated field-scale trials remains limited, and pomegranate-derived preparations should currently be considered complementary components of integrated disease-management programmes rather than direct replacements for registered bactericides and fungicides. The recovery of pomegranate residues may also support local circular-bioeconomy models through the production of botanical pesticides, soil amendments and biofertilizer candidates. Nutrient-fortified pomegranate peel powders have been evaluated as recycled agricultural inputs, but their effects were strongly dose-dependent. Low concentrations were compatible with seed germination and, under some soil-application conditions, promoted plant development, whereas high concentrations of untreated peel powder caused marked phytotoxicity [294]. This dose dependence is particularly relevant because tannins, phenolic acids and other allelopathic or antimicrobial constituents responsible for pathogen suppression may also interfere with seedling development, nutrient availability and non-target microbial communities. Composting, fermentation, nutrient fortification or controlled extraction may reduce phytotoxicity and improve the agronomic value of the residues, but these processes require systematic optimization for each crop and soil type. An emerging perspective is the microbiome-directed use of pomegranate residues. Their polysaccharides, pectins and polyphenols could act as substrates or selective signals in the rhizosphere. Comparable prebiotic-like modulation has been observed for root-secreted coumarins, which interacted with the *Arabidopsis* microbiota to improve iron nutrition [295]. However, direct evidence for pomegranate remains lacking, and dose-controlled soil studies integrating plant performance, microbiome profiling, and soil-enzyme analyses are needed to assess the enrichment of plant growth-promoting rhizobacteria (PGPR) and changes in nutrient cycling.

### 6.5. Advanced Formulations and Delivery Systems

Advanced delivery systems have been investigated to improve the stability, bioaccessibility, and antimicrobial performance of pomegranate-derived bioactives. Microencapsulation by spray drying and nanoencapsulation of pomegranate extracts have been applied to functional foods and active food coatings [296,297], while double emulsions, biopolymer-based particles, and nano-phytosomes have been explored to protect pomegranate polyphenols and modulate their release [14,298,299]. Recent evidence further supports the potential of encapsulation for preserving both the chemical and antimicrobial functionality of pomegranate peel extracts. In particular, alginate-based ionic gelation has been shown to provide effective encapsulation of hydrolysable tannin-rich peel extracts, improving their stability and release compared with spray-dried formulations. The resulting encapsulated extract retained antibacterial activity against *Staphylococcus aureus* when incorporated into a food matrix [300]. These findings are particularly relevant to food applications, where delivery systems may simultaneously protect phenolic constituents and facilitate their controlled release at the site of application. Although these approaches are promising, most available evidence remains at the laboratory or formulation stage, and further studies in realistic food or biological matrices are required to establish whether encapsulation provides reproducible advantages over the corresponding non-encapsulated pomegranate preparations.

### 6.6. Industrial Scale-Up and Market Prospects

Pomegranate by-products offer commercial opportunities in functional foods, nutraceuticals, cosmetics, natural preservatives and animal nutrition. Peel powders and extracts have already been incorporated into meat, dairy and bakery products to improve antioxidant capacity, microbial stability and nutritional value, although excessive concentrations may negatively affect colour, flavour and consumer acceptability [56,301]. Pomegranate-derived polyphenols, oils and polysaccharides are also promising ingredients for supplements, dermatological products and personal-care formulations. In aquaculture, fermented peel polyphenols have improved immunity, antioxidant responses and resistance to bacterial infection in shrimp, supporting their potential as functional feed additives [302]. Industrial valorization would benefit from integrated biorefineries capable of sequentially recovering polyphenols, pectin, seed oil, fermentable sugars and biofuels from the same biomass. Solvent-free hydrothermal processing has enabled the combined recovery of pectin and punicalagin-rich phenolic fractions, followed by bioethanol production from the residual material [303]. Similarly, zero-discharge process design and life-cycle assessment have highlighted the environmental potential of sequential pomegranate-peel valorization [304]. Local-scale enzymatic biorefineries have also generated almost entirely waste-derived emulsion prototypes for food and cosmetic applications, illustrating a possible short supply-chain model for small processing industries [305]. Although these studies demonstrate the potential for pomegranate by-product valorization beyond the laboratory scale, further translation will require reproducible extract composition, scalable processing, safety assessment, and validation of functional and antimicrobial efficacy in realistic application systems.

## 7. Challenges and Future Perspectives

Despite the broad biological and applicative potential of *Punica granatum* L., several limitations still hinder the comparability of experimental findings and their translation into standardized products, as summarized in Table 5.

A major limitation is the intrinsic variability of pomegranate-derived matrices. Cultivar, geographical origin, agronomic conditions, ripening stage and plant fraction strongly influence phytochemical composition [306,307]. Additional variability arises from extraction solvents, processing conditions and analytical procedures. Future studies should therefore provide complete botanical and agronomic information and combine extraction yield with chromatographic fingerprints and quantitative marker compounds [308,309]. Microbiological evidence is also difficult to compare because inoculum density, culture media, exposure time and endpoint definitions vary considerably. Standardized MIC, MBC, time–kill, MBIC and MBEC procedures, together with appropriate solvent and matrix controls, are needed to improve reproducibility [310]. Comparative testing of crude extracts, fractions, reconstructed mixtures and purified compounds would also help distinguish the effects of individual molecules from synergistic or antagonistic interactions within the phytocomplex [183]. Another major gap is the limited transition from in vitro activity to realistic applications. Moreover, promising biological activity should not be equated with immediate practical applicability. Translational feasibility depends on whether the effective concentration can be realistically achieved in the intended setting, whether activity is retained in complex biological or technological matrices, and whether adequate selectivity and safety can be demonstrated. Consequently, evidence obtained from simplified in vitro systems should be regarded primarily as proof of concept until confirmed under application-specific conditions. Validation in food matrices, animal models, crops and humans remains considerably less extensive than laboratory evidence [237,290,291]. Greater attention should also be given to bioaccessibility, pharmacokinetics and microbiota-dependent metabolism, since the conversion of ellagitannins into urolithins varies among individuals and may influence biological efficacy [311]. Safety evaluation should distinguish conventional fruit-derived products from concentrated peel, leaf or root extracts, purified compounds and nanoformulations. Although some standardized extracts have shown favourable toxicological profiles, evidence on chronic exposure, drug interactions, reproductive safety, environmental effects and impacts on probiotics and commensal microorganisms remains limited [312]. Delivery systems may improve stability, bioavailability and controlled release, but carrier toxicity, nanoparticle fate, storage stability and pilot-scale reproducibility require further assessment [14,296]. Finally, industrial translation depends not only on biological efficacy but also on reliable biomass supplies, scalable processing, regulatory classification and economic feasibility. Integrated biorefineries and cascading recovery strategies may increase the value obtained from pomegranate residues, but energy requirements, solvent recovery, seasonal availability and transport may reduce their overall sustainability [304,305]. Future development should therefore integrate efficacy, safety, process engineering, life-cycle assessment, techno-economic analysis and regulatory planning within a multidisciplinary One Health framework [313].

## 8. Conclusions

*Punica granatum* L. represents a highly versatile source of bioactive compounds distributed across the fruit and several underused plant fractions, including peel, seeds, leaves, and flowers. Their phytochemical diversity supports a broad spectrum of biological activities, particularly antioxidant, anti-inflammatory, antibacterial, antifungal, antibiofilm, and microbiota-modulating effects. Sustainable extraction and microbial bioprocessing strategies further enhance the value of pomegranate biomass by enabling the recovery and transformation of functional metabolites within circular production models. Of particular relevance is the activity of pomegranate-derived preparations against multidrug-resistant microorganisms and their potential to complement conventional antimicrobials through synergistic or resistance-modifying effects. These properties suggest potential applications in food preservation, biomedicine, nutraceuticals, animal nutrition, veterinary practice, agriculture, aquaculture, and environmental biotechnology. Despite the broad range of proposed applications, most available evidence remains preclinical or derives from controlled in vitro studies. Therefore, the applicability of pomegranate-derived preparations should be interpreted cautiously, particularly when biological activity is observed at concentrations that may not be realistically achievable in vivo. Translation into practical use will require standardized composition, reproducible biological activity, appropriate safety assessment, stability, and validation of effective concentrations in application-specific in vivo, clinical, food, veterinary, agricultural, or technological models. The level of translational maturity should also be considered separately for each application, since evidence supporting topical, food-contact, or surface-based uses cannot be directly extrapolated to systemic or therapeutic applications. Overall, the available evidence indicates that pomegranate should be regarded not simply as a functional food, but as a multifunctional biological resource connecting phytochemistry, microbiology, biotechnology, and sustainability. Its future practical value will depend on the ability to convert this compositional and functional diversity into reproducible, selective, safe, and application-specific preparations.

## Figures and Tables

**Table 5:** Main challenges, current knowledge and future research priorities for the valorization of pomegranate-derived extracts and compounds.

Challenge	Current Knowledge	Remaining Gap	Future Research Priority	References
Raw-material variability	Composition depends on cultivar, origin, maturity and plant fraction.	Poor batch comparability.	Define botanical metadata and marker ranges.	[306,307]
Extract standardization	Green and conventional methods recover different phytochemical profiles.	Non-uniform extraction and analytical protocols.	Report yield, fingerprints and quantified markers.	[308,309]
Microbiological methods	Antimicrobial and antibiofilm effects are widely reported.	Variable inocula, media and endpoints.	Standardize MIC, MBC, MBIC, MBEC and time–kill assays.	[310]
Active components	Crude extracts, fractions and purified compounds may be effective.	Matrix interactions remain unclear.	Compare extracts and reconstructed mixtures under identical conditions.	[183]
Translational validation	Initial food, agricultural and human studies are available.	Few in vivo, field and clinical studies.	Use realistic models and adequately powered trials.	[237,290,291]
Bioavailability	Ellagitannins are converted into microbiota-derived urolithins.	Strong interindividual variability.	Integrate pharmacokinetics, metabolomics and microbiome profiling.	[311]
Safety and delivery	Some extracts show favourable safety; encapsulation improves stability.	Limited chronic, carrier and non-target safety data.	Assess long-term exposure, microbial selectivity and carrier fate.	[14,296,312]
Industrial feasibility	Biorefineries enable cascading recovery of valuable fractions.	Costs, energy use, seasonality and solvent recovery.	Perform pilot-scale, life-cycle and techno-economic assessments.	[304,305]
Regulation and One Health	Applications span food, health, animal and agricultural sectors.	Fragmented regulatory and cross-sector evidence.	Plan sector-specific approval within a One Health framework.	[309,313]

Abbreviations: MBC, minimum bactericidal concentration; MBEC, minimum biofilm eradication concentration; MBIC, minimum biofilm inhibitory concentration; MIC, minimum inhibitory concentration.

## Data Availability

No new data were created or analyzed in this study. Data sharing is not applicable to this article.
